# Explainable AI for Well-Being Prediction From Lifestyle Data: 2-Study Design

**DOI:** 10.2196/88750

**Published:** 2026-05-08

**Authors:** Flore Vancompernolle Vromman, Corentin Vande Kerckhove, Joël Gagnon, Camille Pelletier, Yannick Dufresne, Simon Coulombe

**Affiliations:** 1 Louvain Research Institute in Management and Organizations, UCLouvain, Place de l'Université 1, Louvain-la-Neuve, Wallonia, 1348, Belgium, 32 479251700; 2 Beneva Research Chair in Mental Health, Self-Management, and Work, Quebec City, QC, Canada; 3 Industrial Relations Department, Université Laval, Quebec City, QC, Canada; 4 Department of Political Science, Université Laval, Quebec City, QC, Canada; 5 CERVO Brain Research Centre, Quebec City, QC, Canada; 6 VITAM – Sustainable Health Research Centre, Quebec City, QC, Canada; 7 Centre d'études et d'interventions en santé mentale, Quebec City, QC, Canada; 8 Centre for the Study of Democratic Citizenship, Montreal, QC, Canada

**Keywords:** well-being, XAI, interpretable models, lifestyle predictors, explanations, human-centered AI, explainable artificial intelligence

## Abstract

**Background:**

Well-being is a cornerstone of public health and social progress; yet, its determinants are multifaceted and dynamic. As behavioral data become increasingly available and artificial intelligence (AI) systems gain prominence, scalable assessments of well-being are becoming more feasible. However, to be useful in practice, such systems must remain understandable to the people they aim to support. Explainable AI is therefore essential to foster trust and enable reflection.

**Objective:**

This research aimed to investigate (1) the extent to which modifiable lifestyle and contextual factors can predict subjective well-being, and (2) how different explanation modalities influence users’ satisfaction when interpreting AI-generated well-being feedback.

**Methods:**

We conducted a 2-stage, application-grounded investigation. First, we developed a parsimonious regularized linear model using a small set of lifestyle-related predictors to estimate individual well-being. Second, we experimentally compared multiple explanation modalities (visual, interactive, textual, quantitative, and population-comparison) against a no-explanation control to evaluate how each format shapes end users’ satisfaction with the AI-generated assessment.

**Results:**

Across conditions, providing any explanation increased users’ satisfaction relative to the no-explanation control in the final sample (n=1252 participants). Visual (B=0.915, SE 0.077; *P*<.001) and interactive (B=0.914, SE 0.076; *P*<.001) explanations produced the highest satisfaction scores, while textual (B=0.850, SE 0.076; *P*<.001) and quantitative (B=0.782, SE 0.077; *P*<.001) formats also showed strong positive effects. Population-comparison (contextual) feedback yielded a smaller effect (B=0.218, SE 0.077; *P*=.005) and was consistently the least preferred and least effective at conveying why the model produced a given assessment.

**Conclusions:**

The findings suggest that well-being tools should combine simple, interpretable models with visual or interactive explanations that foreground actionable behavioral levers rather than emphasizing population norms. These insights offer design guidance for deploying explainable AI in well-being tools to support user satisfaction.

## Introduction

### Background

Well-being is increasingly recognized as a central component of human health and has direct implications for individual, organizational, and societal functioning. It extends beyond the absence of illness, also encompassing positive aspects, for example, positive emotions, meaning in life, that contribute to making life worth living [1]. High levels of well-being are associated with better physical health, stronger relationships, greater work performance, and lower risk of developing mental health disorders [2].

As societies grapple with rising rates of stress, burnout, and mental illness [3-6], monitoring and supporting well-being has become a pressing concern for public health systems [7-11]. Well-being is dynamic and influenced by a broad set of factors that range from socioeconomic status and environmental stressors to personal habits and daily routines. Among these, lifestyle behaviors, such as sleep quality, physical activity, nutrition, and social engagement, have emerged as especially significant because they are modifiable and within individuals’ control [12,13].

Research in psychology and behavioral science increasingly shows that improving these aspects of lifestyle could lead to meaningful improvements in both short- and long-term mental health outcomes [13-17]. However, these insights are rarely translated into user-facing feedback, and many individuals remain unaware of how their behaviors impact their psychological state [18,19]. Therefore, there is a need to raise population awareness with tools that can help take action to improve one’s well-being.

This is where artificial intelligence (AI) can play a transformative role [18]. By analyzing behavioral pattern data across large populations, AI models can identify early signs associated with poor well-being and generate personalized insights based on lifestyle data [20,21]. These predictive models offer a valuable opportunity to increase people’s literacy with regard to well-being (eg, awareness of their own well-being and knowledge about how to improve it) by providing personalized information and guidance directly to users.

While well-being–related AI predictive models hold great promise for raising awareness and supporting self-reflection [22,23], their practical value depends on more than just statistical accuracy [20]. For individuals to trust and interpret AI-generated feedback, they must be able to grasp how the model arrives at its conclusions [24]. In other words, transparency and explainability are not just technical considerations; they are essential for fostering trust and supporting appropriate engagement with such tools [25].

In this paper, we examine how explainability can enhance the transparency and user trust of AI systems designed to predict well-being from lifestyle data. Our approach unfolds in 2 stages. First, we develop a supervised machine learning (ML) model that predicts individuals’ well-being scores from modifiable lifestyle factors in a large, population-representative dataset. Second, we conduct an online experiment to examine how different explanation modalities affect users’ satisfaction with the well-being prediction generated by our system. By integrating model development with user-centered evaluation, this work seeks to clarify how lifestyle data can inform well-being prediction, and how the design of explanations shapes users’ satisfaction.

### Conceptualizing Well-Being

Contemporary frameworks recognize that well-being extends beyond the absence of disease or distress, reflecting instead the presence of positive emotions and functioning in life [26,27]. With regard to the emotional aspects, it refers to individuals’ affective experiences and judgments of life satisfaction [28]. With regard to the psychological and social aspects, it refers to one’s perceptions that they function well at the individual level on dimensions such as self-acceptance, mastery, autonomy, and growth, and at the collective levels, on dimensions such as social integration and social contribution. Keyes [27] proposed a, now well-validated, positive mental health framework that shows how these various aspects are all interrelated parts of well-being, yet distinct from one another [27,29-31], and distinct from indicators of mental health issues, such as depression and anxiety [26].

The determinants of well-being extend beyond dispositional traits and encompass a broad set of modifiable lifestyle, social, and contextual factors. These dimensions are particularly relevant to public health and behavioral science approaches that conceptualize well-being as a dynamic outcome of the interactions between daily habits, social relationships, and living conditions [16]. In line with this perspective, this study focuses on behavioral and contextual variables that are empirically supported and, in several cases, actionable, such as lifestyle habits and social engagement, while also accounting for sociodemographic characteristics that may shape these behaviors.

Well-being can vary across sociodemographic groups, but effects are often modest and context-dependent. Patterns by age may differ across cohorts and measurement periods [16,32-34], and gender differences are typically small [35,36] and often disappear when controlling for covariates, such as income and health [37]. Associations with parenthood are mixed and appear to depend on contextual factors [38-42]. Ethno-racial differences in well-being are complex and intertwined with socioeconomic and structural inequalities [43-47].

Regarding lifestyle behaviors, past research has demonstrated that they play a critical role in predicting well-being. Notably, physical activity has been consistently associated with greater positive affect, vitality, and life satisfaction, as well as reduced symptoms of depression and anxiety [48]. Similarly, adequate sleep quality and duration are strong correlates of well-being [49]. Healthy dietary habits have also been associated with higher levels of well-being. Diets rich in fruits, vegetables, and whole grains contribute to improved mood, energy, and resilience, whereas poor nutrition and substance use (eg, excessive alcohol or tobacco consumption) are negatively associated with mental health outcomes [16]. Other lifestyle activities, such as spending time in nature and engaging in meaningful leisure pursuits (ranging from hobbies to volunteering), are consistently related to greater emotional balance, reduced stress, and enhanced purpose in life [14,15]. Collectively, these findings underscore that well-being is not only influenced by intrapersonal factors but is deeply rooted in everyday behavioral choices.

Social relationships and community involvement are also central to the experience of well-being. Across more than 150 countries, indicators of social connection and perceived support were found to be positively associated with positive life evaluation and positive affect, even after controlling for sociodemographic and contextual variables [50]. Moreover, empirical research indicates that individuals who report high levels of social connectedness and engagement in their communities tend to experience higher levels of life satisfaction, purpose, and positive affect [49]. Strong and supportive networks are not only associated with greater happiness and purpose in life but also with improved health outcomes and longevity [51,52]. Supportive work environments also play protective roles in well-being. Social support at work has been linked to reduced burnout, greater motivation, and enhanced overall well-being [16]. Likewise, participation in community-oriented activities such as volunteering, civic engagement, or informal helping behaviors strengthens one’s sense of meaning and contribution [45].

### AI for Well-Being

Although the literature identifies a wide constellation of determinants of well-being, most existing studies examine only a limited subset of these factors at a time [53]. This means that lifestyle, social, contextual, and sociodemographic influences are often analyzed in isolation, within separate datasets or specialized models. As a result, current approaches rarely offer a holistic view of how multiple modifiable behaviors jointly contribute to well-being, limiting their ability to deliver personalized guidance for individuals. This gap highlights the need for integrated models that incorporate diverse lifestyle and contextual variables within a single statistical framework.

Many determinants of well-being are measurable at large-scale levels via surveys, smartphone apps, wearables, etc. It creates opportunities to deploy AI tools to monitor behaviors, support decision-making, and predict outcomes relevant to mental health and well-being [54-56]. AI broadly refers to computational methods that perform tasks replicating or supplementing human intelligence (eg, perception, pattern recognition, prediction, and decision support) [57]. A major subset of AI is ML, that is, machines that learn patterns from data [57]. ML may be used to predict an outcome (eg, well-being) from input factors (eg, lifestyle and sociodemographics).

In the well-being literature, ML is used in different predictive settings, including both regression (eg, predicting continuous life satisfaction or well-being scores [40,49]) and classification (eg, predicting stress levels or risk groups [58-60]). Across these tasks, researchers have implemented a variety of model classes, ranging from traditional statistical models, such as linear and regularized regression, to more flexible nonlinear approaches, including tree-based models and neural networks [61,62]. These model classes differ in their inductive biases and representational capacity—linear models impose additive and approximately linear relationships between predictors and outcomes, whereas tree-based and neural models can capture complex, nonlinear interactions in high-dimensional behavioral data. In applied work, this variety of models has facilitated the use of multimodal predictors to model well-being outcomes. Oparina et al [59] showed that diverse variables, including lifestyle behaviors, health status, socioeconomic information, and digital behavior, can be used to predict well-being. Wang et al [58] used smartphone signals (sleep, movement, and social activity) collected during daily life to estimate stress, mood, and well-being, demonstrating that passive, everyday behaviors carry useful information. Khan et al [40] used ML on structured questionnaires to predict life satisfaction and showed that lifestyle and health-related answers ranked among the most influential predictors.

However, despite these advances, most existing ML studies focus primarily on emotional aspects of well-being. For example, Oparina et al [59] predict only emotional well-being, and Margolis et al [49] also concentrate mostly on emotional indicators, with only limited coverage of psychological dimensions. As a result, current predictive approaches do not fully capture the multidimensional nature of well-being [30,31]. This gap highlights the need for models that can predict well-being as a broader construct integrating emotional, psychological, and social components.

### From AI to Explainable AI

While AI offers promising results in predicting well-being, the ability of such systems to generate impact in real-world settings depends on more than predictive accuracy [63]. In sensitive domains, such as well-being, users’ trust and understanding are essential for ML to have a meaningful impact in practice [20,64]. However, users’ trust cannot be achieved solely through a high accuracy of the ML system; it also requires that users understand how and why the system generates its outputs [21,24]. This is where the explainability of the model predictions becomes crucial, as it helps end users understand and trust model predictions [25]. Without clear explanations, users may misinterpret results, disregard useful insights, or lose confidence in digital tools [25,65].

Explainable AI (XAI) has been widely studied in recent years. It refers to making the outputs of ML systems understandable to humans, particularly to the end users of those systems [24,25,63,66-68]. Explanations may be characterized as global when they relate to the model’s behavior, or local when they refer to a specific prediction produced by the model [66,68-70]. A large class of XAI methods focuses on explaining how an ML model maps inputs to outputs and can therefore be described as model-centric approaches. These methods can be grouped into several families, such as feature-based explanations (features’ influence on model predictions), rule-based explanations (if-then decision rules linking conditions to predictions), example-based explanations (similar or representative instances from a reference set), and counterfactual explanations (input features changes required to yield a different, typically desired, outcome) [66,71-76]. Besides these model-centric approaches focusing on how an ML system maps inputs to outputs, data-centric approaches of XAI focus on transparency about the training data used by the system (eg, distribution, coverage, or missingness of training data) [74,77,78], since models trained on historical data can reflect or reinforce biases [69].

Beyond the distinction between explanation families, the explainability of an ML system is closely tied to the choice of the underlying prediction model. In practice, models vary widely in their complexity and flexibility, giving rise to a well-known trade-off between predictive performance and ease of understanding [79]. More flexible models can capture complex and nonlinear relationships in high-dimensional behavioral data but are harder to analyze, whereas simpler models impose stronger structural assumptions that facilitate inspection and understanding at the cost of expressive power [80]. This trade-off is often described as a distinction between intrinsically interpretable models and black-box models [81]. Intrinsically interpretable models, such as linear or regularized regression and simple rule-based models, expose structures that can be directly examined, while more complex models, such as tree-based ensembles or neural networks, are typically treated as black boxes and require post hoc explanation methods [82], a distinction that is particularly salient in sensitive domains, such as well-being, where model outputs are intended to support human decisions.

The choice among explanation approaches depends on the domain, the users, and the intended purpose of the system [83]. In this study, we focus primarily on feature-based explanations as a way to support the interpretation of model predictions in the context of well-being. Feature-based explanations provide a direct and concise representation of how input variables contribute to individual predictions, making them a practical choice for communicating model behavior. Feature-based explanations allow users to directly identify which behavioral dimensions are driving their predicted score in a concise and personalized manner [84]. This focus does not preclude the relevance of other explanation paradigms, but they were not prioritized here for specific reasons. Example-based explanations rely on comparisons with other individuals, which raises concerns about representativeness and privacy in a sensitive health-related domain. Rule-based explanations require expressing model behavior through discrete if-then statements, which can become complex or brittle for a continuous and multifactorial construct such as well-being. Counterfactual explanations are well-suited for exploring hypothetical changes, but in the absence of causal models, they remain associational and risk being overinterpreted as prescriptive advice. Finally, data-centric explanations are essential for transparency about training data and model scope, but they do not explain individual predictions and therefore do not directly support personalized feedback.

Regarding feature-based explanations, achieving explainability involves 2 steps—quantifying how input features contribute to model predictions, and communicating these explanations to users in a way that supports trust and understanding [25,70,83]. While the first step is well-studied in the XAI literature, the second has historically received less attention [83,85-87]. Human-centered XAI addresses this gap by grounding explanation design and evaluation in users’ goals, context, and practical needs [88-92]. In the following, we examine these 2 complementary aspects of explainability.

### Feature Weights for Feature-Based Explanations

A central class of model-centric explanation methods consists of feature-based explanations, which aim to characterize how input variables contribute to a model’s predictions. In the literature, this idea is referred to under various terms, including feature weights, feature importance, or feature attribution, but the underlying objective is the same: to quantify the influence of each input feature on the predicted outcome [66]. Such quantities provide a compact and intuitive summary of how a model uses its inputs and form the technical basis for many user-facing explanation interfaces.

The way feature weights are obtained depends on the type of prediction model used. For linear or regularized regression models, feature weights do not need to be approximated; they are directly given by the estimated regression coefficients, which indicate the direction and magnitude of each feature’s effect on the prediction [24]. For more complex models, such as tree-based ensembles or neural networks, feature weights must be estimated rather than read directly from the model. In this setting, post hoc attribution methods approximate the local or global influence of input variables by probing the model’s response to controlled variations of the inputs. Prominent examples include Shapley Additive Explanations [84], which compute feature attributions based on concepts from cooperative game theory, and Local Interpretable Model-agnostic Explanations [93], which fit local surrogate models around individual predictions. These methods provide feature-level attribution scores that summarize how input variables contribute to a specific prediction or to the model’s behavior more broadly [68], albeit as approximations rather than exact quantities.

This distinction highlights that the choice of prediction model has direct implications for how feature-based explanations are constructed. Intrinsically interpretable models allow feature weights to be obtained directly and used as explanations without an additional approximation step. However, such models often impose stronger assumptions on the relationship between inputs and outputs and may be less effective at capturing complex, nonlinear patterns in high-dimensional data [24]. Conversely, more flexible black-box models can achieve higher predictive performance but require post hoc methods to approximate feature weights. This 2-stage process introduces additional sources of uncertainty, as both the prediction model and the explanation method may be imperfect, and the resulting feature attributions remain approximations that can omit important aspects of the model’s behavior or even mask problematic patterns [79,94].

### From Feature Weights to Explanation Modalities

While much of the XAI literature has focused on quantifying feature weights for explanations (first step), the translation of those mathematical or technical explanations into formats that users can understand has historically received comparatively less attention [66,69,83,85-87,95]. In line with human-centered XAI, users should be involved throughout the explanation design [88,90,92]. This step is essential because, if neglected, users may misinterpret technical details, develop incorrect conclusions, or build too high confidence in the model [83,86,94,96,97]. Therefore, the way explanations are presented can strongly influence how users interpret the model’s output, their level of trust in it, and whether they are motivated to act on the feedback received [66,74,78,85,91,94].

One approach is to transform the feature weights into textual explanations for users. For example, in the mental health domain [98], explain outputs of a recommender system through semantic narratives linked to adverse childhood experiences. Recently, large language models (LLMs) have been proposed as a practical way to support this translation step [99]. For example, LLMs may be used to transform technical XAI outputs into user-readable narrative explanations (eg, studies by Martens et al [95] and Zytek et al [100]). Beyond one-shot narratives, LLMs are also used to enable conversational explanations, where users can ask follow-up questions and request alternative views of the same prediction (eg, studies by Nguyen et al [101], Slack et al [102], and Samimi et al [103]).

Beyond textual explanations, other works in the mental health domain have explored visual formats such as bar plots or radar charts to make feature weights more tangible [21,98]. These visual representations aim to reduce cognitive load and support intuitive comparisons between factors, thereby helping users better understand which aspects of their profile are most strongly associated with the model’s predictions [103,104].

Explanations can also be delivered through interactive interfaces that allow users to actively explore a model’s behavior by manipulating inputs and observing the resulting changes in predictions [105]. Such interactive explanations shift the user from a passive recipient of information to an active participant in sense-making, supporting exploratory and iterative reasoning about the model [103,105,106]. This interaction paradigm is closely related to “what-if” explorations or counterfactual analyses discussed in the XAI literature, in that users examine how predictions vary under hypothetical input changes. In the mental health domain, Joyce et al [25] present an interactive interface that allows clinicians to adjust patient factors (eg, symptoms or risk indicators) and immediately see how the model’s predicted risk changes.

### Evaluating Explanations

A major challenge in XAI is the evaluation of explanations. In practice, explanation quality is often assessed using technical metrics, such as faithfulness to the prediction model (ie, do they accurately reflect how the model makes predictions), or stability-consistency (ie, small input changes yield small explanation changes) [70,107]. While these metrics are useful to assess explanations from a technical perspective, they remain “functionally-grounded evaluation” [70], since they assess explanations only at the model level, without capturing whether users actually find them understandable or useful in practice [108]. Therefore, evaluation should consider not only technical properties but also how explanations are presented to end users [75].

It has motivated “human-grounded evaluation,” where human participants judge explanations through simplified or abstracted tasks, often outside real use contexts and frequently with participants who are not the intended end users [70,94,109]. Several standardized instruments have been developed to support such studies [109-112]. For instance, Hoffman et al [109] introduced the Explanation Goodness Checklist, primarily intended for experts to assess completeness and coherence. While useful for early-stage testing, these evaluations can have limited ecological validity because tasks are often artificial and the resulting judgments remain proxies rather than the lived experience of actual end users.

In contrast, application-grounded evaluation assesses explanations directly with intended users performing the intended tasks in context [70]. This approach offers the strongest ecological validity, but remains rare due to the difficulty of recreating real deployment conditions [70,110,113]. Hoffman et al [109] also developed the Explanation Satisfaction Scale, designed for end users to rate perceived understanding, sufficiency, usefulness, and trust of explanations. In well-being contexts, where outcomes are subjective, personal, and context-dependent, only individuals themselves can meaningfully judge whether explanations are clear, trustworthy, and actionable [88,91,92,108]. For this reason, application-grounded evaluation is essential, as technical metrics or proxy human assessments cannot capture the experiential relevance of explanations [25,65,70,94,109].

### Research Aims

Despite growing attention to XAI, few studies have examined how explanation modalities for well-being prediction systems perform when evaluated directly with end users in realistic contexts. Previous work has mainly focused on technical aspects of model transparency, leaving open how different explanation modalities influence users’ trust and understanding of AI-generated feedback [66,83,85-88,91].

**Figure 1.**
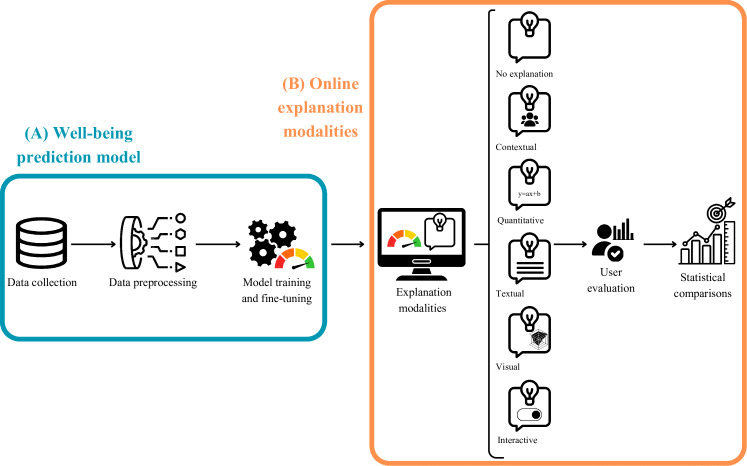
Pipeline overview showing (A) the development of the well-being prediction model and (B) the design of online explanation modalities.

To address these gaps, this paper adopts a 2-stage research design summarized in Figure 1. Study 1 develops a supervised ML model that predicts individuals’ well-being scores from lifestyle and contextual variables in a large, population-representative sample. Study 2 experimentally explores how distinct explanation modalities affect users’ satisfaction with the model’s feedback.

The research is guided by 3 questions: first, to what extent can lifestyle and contextual factors predict subjective well-being; second, how do different explanation modalities shape users’ satisfaction with AI-generated feedback; and third, whether individual characteristics influence how explanation modalities are received. Together, these studies clarify how explainability can bridge the gap between algorithmic prediction and human interpretation.

The remainder of the paper follows this 2-stage design, presenting study 1 and study 2 in sequence, each with its Methods and Results, followed by a general Discussion.

## Study 1: Well-Being Prediction Model

Study 1 implements the first stage of our pipeline (Figure 1), developing a prediction model that estimates well-being scores from participants’ lifestyle data.

### Methods

#### Participants

The survey for study 1 was designed and administered online using the Qualtrics platform, which allowed for standardized presentation of questions and secure data collection. Data collection was conducted through the Leo Web Panel, a Canadian panel of participants managed by the Leger survey firm. Participants were recruited from the panel’s database and compensated with reward points for their time. To ensure the sample was representative of the Canadian population, we implemented quota sampling based on proportions derived from Statistics Canada census data [114]. Quotas were established for geographic region, age, gender, and immigration status, ensuring proportional representation across Canadian provinces and territories, with an appropriate distribution matching the adult population age structure, representation reflecting national gender demographics, and inclusion of both Canadian-born and immigrant populations in line with national statistics. Data collection continued until predetermined quotas for each subgroup were met, resulting in a final sample of 2058 participants. This quota-based approach enhances the generalizability of our findings to the broader Canadian adult population while maintaining sufficient sample size for ML model development.

#### Ethical Considerations

This study received ethical approval from the Université Laval Research Ethics Board (approval 2022‐255 phase II, approval date: November 6, 2023). Participants were recruited through the Léger Opinion panel and were compensated with reward points according to survey length (500 points, equivalent to CAD $0.50 [US $0.37] per 5 minutes), redeemable for cash, gift cards, or donations. All participants provided informed consent before participating in the survey, and data were collected and stored in accordance with privacy regulations and institutional guidelines for human subjects research.

#### Questionnaire

The questionnaire was designed to capture a comprehensive profile of lifestyle behaviors, social engagement, sociodemographic characteristics, and well-being. Drawing on the literature linking lifestyle factors to mental health outcomes [12-16,48,49,115,116], we constructed a 280-item survey instrument organized into several thematic domains. The selection of questionnaire items was informed by previous research demonstrating their relevance to well-being prediction [1,40,117,118], with particular attention to modifiable lifestyle factors that individuals can feasibly change and act on, while also considering contextual factors that are less amenable to change (eg, sociodemographics). Importantly, all items were either validated as single-item measures in previous studies or selected from longer validated instruments based on their theoretical relevance and previous factor-analytic evidence identifying them as the most representative indicators of the underlying construct.

Workplace support, volunteer activities, and relationship quality were incorporated as key indicators of social engagement, reflecting core psychosocial dimensions of well-being [119-121]. To capture lifestyle behaviors, we included items assessing sleep patterns, physical activity, dietary habits, alcohol and tobacco use, screen time, and daily routines, factors repeatedly shown to shape mental health and well-being. Sociodemographic variables, including age, gender, immigration status, and region, as well as self-reported health conditions, were collected to enable population-representative modeling and control for confounding factors. To ensure data quality, we also embedded 3 attention check items throughout the questionnaire.

For the assessment of well-being, we used the Mental Health Continuum-Short Form (MHC-SF [30,31]), a 14-item self-report questionnaire measuring 3 dimensions of positive mental health. The scale assesses emotional well-being (3 items; eg, “How often did you feel happy?”), psychological well-being (6 items; eg, “How often did you feel that you had warm and trusting relationships with others?“), and social well-being (5 items; eg, “How often did you feel that you belonged to a community?”). Respondents rate how frequently they experienced each indicator of well-being during the past month on a 6-point Likert scale ranging from 0 (never) to 5 (every day). Items are summed to create a total well-being score ranging from 0 to 70, with higher scores indicating greater well-being. The MHC-SF has demonstrated good internal consistency across multiple studies, with Cronbach α values ranging from 0.74 to 0.89 for the total scale [31,122]. In this study, the Cronbach α for the total scale was 0.93.

#### Data Analysis Strategy

The goal of study 1 was to develop a supervised learning model capable of predicting individuals’ well-being scores from a wide range of lifestyle and sociodemographic indicators. The prediction pipeline comprised 3 main stages—feature engineering, feature selection, and model training with cross-validation and hyperparameter tuning. This multistage approach was designed to balance model interpretability and predictive performance while ensuring robustness and generalizability across the sample. All analyses were conducted using Python (version 3.9.9) and standard data science libraries including pandas, scikit-learn, and extreme gradient boosting (XGBoost). To ensure data quality, participants who failed more than 1 of the 3 embedded attention checks were excluded from the analysis.

The first phase of the analysis involved the creation of a comprehensive feature library derived from participants’ responses to the 280-item questionnaire. To ensure that the predictive model focused on lifestyle indicators conceptually distinct from subjective well-being itself, we excluded all items that (1) contributed to the computation of the target variable (well-being score) and (2) required internal evaluative judgments of psychological functioning or well-being. Importantly, the selection of lifestyle indicators was guided by a pragmatic methodological definition developed in consultation with well-being experts on our team. Lifestyle indicators were conceptualized as existing on a continuum of subjectivity, ranging from directly observable or behaviorally anchored activities (eg, volunteering behavior and health care use) to self-reported indicators embedded in individuals’ everyday living and working conditions (eg, workplace support and living environment). Although some retained indicators are self-reported and, thus, perceptual in nature, they focus on concrete behaviors, roles, or contextual conditions that are externally referable and potentially actionable, rather than global assessments of mental or emotional well-being. This refinement step ensured that the model relied on contextualized lifestyle indicators that are interpretable and relevant for potential behavioral or environmental interventions. After applying these filtering criteria, 74 question items were retained from the original 280-item questionnaire for feature construction. To comply with copyright restrictions that preclude reproducing full proprietary scales or subscales, we provide only the 74 items used for feature construction in Multimedia Appendix 1, rather than the full questionnaire. Researchers interested in consulting the complete questionnaire may contact the authors directly. Feature preprocessing procedures are detailed in Multimedia Appendix 2. After preprocessing, the 74 questionnaire items expanded into a feature library comprising 214 engineered features.

Model development and evaluation were conducted using a 5-fold cross-validation procedure, which served as the overarching framework for all preprocessing, feature selection, and model training steps. This design ensured that all data-driven decisions were made using training data only and evaluated on held-out test data.

Within each cross-validation fold, the training partition was used to perform a complete modeling pipeline comprising (1) imputation of missing values, (2) feature scaling, (3) feature selection, and (4) model estimation. Feature selection was performed within each training fold to ensure that the predictive model remained interpretable and practical for deployment while preventing information leakage. Specifically, starting from an initial library of 214 engineered features, feature importance was estimated using only the training data in that fold, and a reduced subset of high-value features was selected before model fitting. The full feature selection procedure, including how feature importance was computed and how the final subset was derived, is described in Multimedia Appendix 2.

The target variable (well-being score) was rescaled between 0 and 100 to facilitate interpretability and comparability across models. For each fold, predictions from the best-performing hyperparameter configuration were generated on the held-out test partition, and model performance was evaluated using mean absolute error (MAE), mean square error (MSE), and the coefficient of determination (*R*²). This multimodel, nested cross-validation design provided a robust estimate of out-of-sample generalization performance, reducing the risk of overfitting and bias during model selection.

### Results

#### Participant Overview

A total of 2058 individuals completed the survey. Of these, 1881 (91.4%) passed at least 2 of the 3 attention checks and were retained for analysis, whereas 177 (8.6%) were excluded for insufficient attention. All subsequent analyses were conducted on the final sample of 1881 participants.

Regarding gender, 965 out of 1881 (51.3%) identified as men, 890 out of 1881 (47.3%) as women, and 26 out of 1881 (1.4%) identified with another gender identity (including nonbinary, queer, agender, or other). In terms of education, 632 out of 1881 (33.6%) had completed college, Collège d'enseignement général et professionnel, or classical college; 566 out of 1881 (30.1%) held a bachelor’s degree; 395 out of 1881 (21%) had a high-school diploma; 226 out of 1881 (12%) held a master’s degree; 34 out of 1881 (1.8%) held a PhD; 26 out of 1881 (1.4%) had completed elementary school; and 4 out of 1881 (0.2%) reported no schooling.

Household income was distributed as follows: US $1-US $30,000 (250/1881, 13.3%), US $30,001-US $60,000 (429/1881, 22.8%), US $60,001-US $90,000 (404/1881, 21.5%), US $90,001-US $110,000 (248/1881, 13.2%), US $110,001-US $150,000 (282/1881, 15%), US $150,001-US $200,000 (156/1881, 8.3%), more than US $200,000 (77/1881, 4.1%), and no income (34/1881, 1.8%).

Parental status indicated that 1403 out of 1881 (74.6%) reported having no children, 248 out of 1881 (13.2%) had 1 child, 162 out of 1881 (8.6%) had 2 children, 51 out of 1881 (2.7%) had 3 children, 9 out of 1881 (0.5%) had 4 children, and 8 out of 1881 (0.4%) reported 5 or more children.

Participants’ age averaged 49.26 (SD 17.99) years, with a median of 49 (IQR 34-64) years and a range of 18‐97 years.

In the final sample, the rescaled well-being score (0‐100) had a mean of 57.31 (SD 21.39) and ranged from 1.43 to 98.57, indicating substantial outcome variability and contextualizing the magnitude of the MAE values reported in the Prediction Model Performance section.

#### Prediction Model Performance

Table 1 summarizes the average results across the 5 folds. As expected, the baseline models underperformed. The mean regressor reached an MAE of 17.8, an MSE of 474.2, and an *R*² of −0.001, indicating that it did not explain any variance beyond the population mean. The random regressor performed even worse, with an MAE of 31.0, an MSE of 1420.4, and a strongly negative *R*² of −1.77, reflecting performance inferior to the mean baseline. These results confirm that simple heuristic predictions failed to capture meaningful patterns in the data.

**Table 1.** Average out-of-sample model performance computed across 5-fold cross-validation splits.

Model	Mean absolute error	Mean square error	Coefficient of determination (*R*^2^)
Mean regressor	17.8	474.2	0.00
Random regressor	31.0	1420.4	−1.77
Ridge regressor^a^	15.0^a^	341.4^a^	0.28^a^
XGBoost^b^ regressor	15.2	359.6	0.24

a Indicates the selected model, achieving the best predictive accuracy.

b XGBoost: extreme gradient boosting.

Among the trained models, the ridge regression model achieved the best predictive accuracy overall, with an MAE of 15.0, an MSE of 341.4, and an *R*² of 0.28, indicating that the model explained approximately one-third of the variance in well-being scores on unseen data. The XGBoost regressor performed slightly worse (MAE 15.2, MSE 359.6, *R*² 0.24), showing that the additional model complexity did not improve predictive performance.

Given the marginal performance difference and the additional complexity of the XGBoost pipeline, the ridge regression model was selected for deployment, offering an optimal balance between accuracy, simplicity, and interpretability. Its moderate *R*² further supports its value as an explainable predictive baseline for subsequent user studies examining explanation quality and behavioral responses.

While cross-validation was used to obtain unbiased estimates of out-of-sample predictive performance, the final ridge regression model was subsequently refit on the full dataset using the same feature selection procedure in order to support stable estimation and interpretation of feature coefficients. This final model was used exclusively for descriptive and explanatory analyses and not for performance evaluation. Applying the feature selection procedure led us to retain 20 questionnaire items, which together expanded into 36 features due to the inclusion of multicategory variables. These items represent the subset of lifestyle, behavioral, and contextual factors that contributed most strongly to predicting well-being scores. The full feature-ranking results and detailed selection process are reported in Multimedia Appendix 2.

Approximately 91.66% (33/36) of the features exhibited very low levels of missingness (≤1%), while 1 categorical feature showed moderate missingness (4.84%). Furthermore, 2 workplace-related variables, reflecting perceived support when facing work-related difficulties and autonomy over taking breaks, showed higher missing-data rates (approximately 43%). This higher missingness is primarily attributable to their conditional relevance, as these items apply only to respondents currently engaged in paid employment and are therefore not applicable to participants who were retired, unemployed, or otherwise not working at the time of the survey. Missing values were handled through imputation applied within each cross-validation training fold as part of the preprocessing pipeline. For the final model refit on the full study 1 dataset, the selected imputation strategy was a k-nearest neighbors imputer (k=5, Euclidean distance), as determined by hyperparameter selection on the full training data. Feature scaling was performed using a MinMaxScaler, and the final ridge regression model used an L2 regularization strength of 4.0, corresponding to the best-performing hyperparameter configuration.

To verify that the 36 engineered features contributed independently to the model, we assessed multicollinearity using variance inflation factors. Variance inflation factor values indicated no meaningful collinearity concerns. Complete results are provided in Multimedia Appendix 3.

#### Most Influential Positive and Negative Predictors of Well-Being

As described in the previous section, we used a linear regression model with L2 regularization to evaluate the predictive importance of variables on the well-being score. The model coefficients serve as indicators [24,68] of the direction and strength of the relationship between each feature and the well-being outcome.

The 5 strongest positive predictors of well-being were related to sleep quality, self-care and autonomy at work, frequency of social activities with friends, satisfaction with one’s living environment, and volunteering behavior. Conversely, the 5 strongest negative coefficients were associated with perceived lack of workplace support, employment in health-related occupations, presence of chronic health conditions, and consulting specific health care professionals for mental health concerns. A complete overview of all standardized coefficients is provided in Multimedia Appendix 4.

## Study 2 - Online Explanation Modalities

### Overview

Building on the predictive model developed in the first stage of the pipeline, we proceeded with the evaluation of explanation modalities. The selected feature set offers sufficient diversity to capture key lifestyle and psychosocial determinants of well-being while remaining feasible for practical online deployment, and the resulting parsimonious model provides an appropriate basis for generating individualized predictions.

Study 2 implements the second stage of our pipeline (Figure 1), by examining how different explanation modalities shape users’ satisfaction with the predicted well-being score. To do so, we conducted a randomized online experiment in which participants were allocated to one of several explanation conditions, enabling direct comparison of formats in terms of explanation satisfaction.

### Methods

#### Participants

For study 2, we built a custom web application in Python (using Flask) to serve as the survey platform. This enabled direct integration of our well-being prediction algorithm from study 1 and live display of predicted well-being scores from participants’ lifestyle answers, something not feasible with standard survey platforms such as Qualtrics.

While study 1 relied on the Leger Panel to obtain a representative Canadian sample for model training, study 2 required a different recruitment strategy due to its interactive, web-based experimental design. Participants were therefore recruited through the Prolific platform. This platform allows precise control over random assignment and ensures smooth interaction with dynamic interfaces, making it more suitable for testing user experience. Although Prolific samples are not fully representative of the Canadian population, previous research shows that data quality and attention levels are high. Moreover, we later examine whether this difference in participant composition could meaningfully influence the study’s findings. Participants recruited on Prolific were redirected to our platform. To match study 1’s population, recruitment was restricted to Canada-based adults. Because Prolific’s quota tooling did not, at the time of data collection, support census-aligned quotas for Canada, we could not enforce sociodemographic quotas at recruitment. Instead, we recorded sociodemographics, conducted balance checks across the six experimental conditions (chi-square for categorical variables; Kruskal-Wallis for age), and included these variables as covariates in the primary analyses to assess any impact on explanation satisfaction. We recruited participants on Prolific until each experimental condition contained at least 200 valid participants (ie, those who passed 2 embedded attention checks).

#### Ethical Considerations

As in study 1, this study received ethical approval from the Université Laval Research Ethics Board (approval 2024‐389 A-1; approval date: July 8, 2025). All participants provided informed consent before participating in the survey, and data were collected and stored in accordance with privacy regulations and institutional guidelines for human subjects research. Participants were paid according to Prolific’s fair-pay guidelines (estimated 15 minutes, £11/hour, ie, £2.75 [US $3.68] per participant).

#### Questionnaire

**Figure 2.**
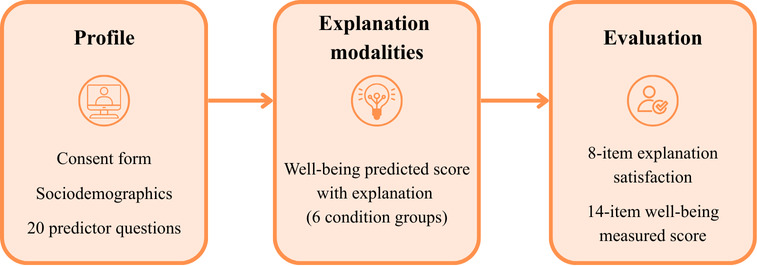
Overview of the questionnaire flow through the online study. Participants first completed the consent form, sociodemographic questions, and the 20 predictor questions (Profile), then viewed their predicted well-being score with one of six explanation conditions (Explanation modalities), and finally answered satisfaction and well-being questionnaires (Evaluation).

Figure 2 presents the questionnaire flow for study 2. The complete questionnaire for study 2 is available in Multimedia Appendix 5.

##### Profile

In the initial “Profile” phase (Figure 2), participants provided informed consent, then completed a brief sociodemographic section (ie, gender, age, education, income, parental status, and immigration status), followed by the 20 lifestyle questions. These 20 questions correspond to those selected from study 1 as the features used by our model to generate the live predicted well-being score.

##### Explanation Modalities

All participants received their predicted well-being score, accompanied by a standardized introductory message describing the model as data-driven. This framing statement, identical across conditions (including the control group), is reproduced in Multimedia Appendix 6. Its purpose was to introduce the model in a neutral and credible manner while avoiding any personalized explanation.

As discussed previously, the presentation format of explanations (ie, the explanation modalities) plays a decisive role in shaping users’ satisfaction with the explanations. Building on this rationale, we designed 4 distinct explanation modalities (ie, quantitative, textual, visual, and interactive), each corresponding to a separate experimental condition, along with 2 additional comparison conditions. One comparison condition served as a control group in which no explanation was provided. The other was a contextual condition, reflecting a common design pattern in well-being and health tools, where users are shown how their score compares to a reference population without any model-based explanation of contributing factors.

The contextual condition was included to contrast model-based explanations with purely comparative, nonexplanatory feedback. The condition was inspired by research emphasizing socially anchored explanations, where individual outcomes in the mental health domain are interpreted relative to comparable populations [25,123,124]. This format situates a user’s predicted well-being score within a broader distribution of peers, enhancing interpretability through social comparison and perceived relevance. To construct this explanation, the distribution of well-being scores was divided into quartiles representing increasing levels of well-being computed from study 1 data. The first quartile comprised scores below 43, the second between 43 and 59, the third between 59 and 74, and the fourth encompassed scores above 74. For each participant, the predicted well-being score was mapped to its corresponding quartile, which was then highlighted in the explanation interface. This approach situates individual predictions within the broader population distribution, enabling users to interpret their results through social comparison rather than model-derived reasoning.

The 4 explanation modalities (ie, quantitative, textual, visual, and interactive) were derived from previous work in XAI and human-computer interaction and correspond to different ways of presenting the same underlying feature-based information derived from the model to end-users.

The quantitative explanation builds on the tradition of feature-importance transparency in XAI [66,85], presenting model reasoning in a numerical form that highlights the key predictors driving the output. This approach favors users who prefer concise, data-oriented information over interpretive or narrative formats. It presented a structured, numeric breakdown of the participant’s predicted well-being score. The explanation displayed the total predicted score alongside a list of the 5 most influential lifestyle factors identified by the ridge regression coefficients. For each factor, the interface specified its contribution in points (computed as the product of the feature coefficient and the participant’s normalized response value) and allowed users to expand a collapsible text panel to view how this contribution was calculated. Additional factors not displayed were summarized at the end of the page to maintain contextual completeness.

The textual explanation draws from research on narrative and linguistic explanations [95,98], which use plain, human-readable text to convey the reasoning behind predictions. Rather than displaying technical metrics, this condition provides a short descriptive paragraph emphasizing behavioral factors and model insights in an accessible and human-centered manner. The explanation provided a narrative counterpart to the quantitative version, removing numerical content to promote interpretability for nontechnical users. The same 5 influential lifestyle variables were described in plain language, emphasizing their relevance to well-being. Each factor was accompanied by a short explanatory paragraph detailing why the variable is typically associated with higher well-being scores. The user’s own responses were embedded in the narrative, and collapsible sections allowed for further elaboration on each factor’s psychological or behavioral importance.

The visual explanation reflects the growing body of work exploring graphical representations of model behavior [21,98]. In this condition, feature importance is depicted through a radar chart accompanied by a brief interpretation generated from model outputs, allowing users to quickly apprehend relative strengths and weaknesses across lifestyle dimensions. The visual explanation displayed a radar chart visualization of the same top features. The chart displayed each factor’s normalized value for the participant, allowing for intuitive comparison across domains (eg, sleep, social activities, and work autonomy). A concise textual summary accompanied the visualization to guide interpretation while avoiding mathematical notation. This multimodal format aimed to support comprehension through both verbal and spatial reasoning channels.

Finally, the interactive explanation extends the idea of counterfactual and exploratory explanations [71-73], enabling users to directly manipulate key input variables and observe real-time updates to their predicted well-being score. The interactive interface is built on the same feature-based explanatory content as our other modalities, but differs in how users access and manipulate this information through direct exploration. This hands-on format encourages exploration of “what-if” scenarios, promoting deeper understanding and engagement with the model’s logic. It was implemented as a dynamic interface where participants could directly adjust their own input values for the most influential predictors. Each modification triggered an immediate recalculation of the predicted well-being score, displayed in real time through a gauge chart. This design allowed participants to actively explore how changes in their responses affected the model’s output, transforming the explanation into an experiential and self-guided interaction rather than a static description.

Together, these 4 explanation modalities represent a range of communication approaches that vary in descriptiveness, level of detail, and interactivity. All model-based modalities—except the baseline and contextual conditions—were built from the 5 most influential predictors identified in study 1, selected for their strong positive contribution to well-being and their suitability for concise, actionable explanations. The procedures used to generate each modality, including how these 5 features were selected and how pregenerated LLM templates were combined with participant-specific numerical values inserted into predefined HTML, are detailed in Multimedia Appendix 7. Interface screenshots for all modalities are provided in Multimedia Appendix 6.

##### Evaluation

To evaluate and compare explanation modalities (refer to “Evaluation” in Figure 2), we used the 8-item scale “Explanation Satisfaction Scale” of Hoffman et al [109]. This scale is designed for end users to rate explanation satisfaction. Items were rated on a 5-point Likert scale and averaged (higher values indicate greater satisfaction). In this study, the Cronbach α for this scale was 0.91.

Because users’ reactions to explanations may depend on how accurate they perceive a model’s output to be [109,125], it is important to account for the discrepancy between predicted and measured well-being when analyzing explanation satisfaction. Participants therefore also completed the 14-item MHC-SF [30,31], as in study 1, to obtain a measured well-being score. This score was used to compute prediction error, which is included as a covariate in the analyses presented in the Data Analysis Strategy section.

Because study 2 involved experimental conditions, we included manipulation checks, administered after the Explanation Satisfaction Scale to avoid influencing satisfaction ratings, to verify that each experimental condition had the intended effect [126-128]. Participants answered 5 manipulation checks (1 per explanation modality) on a 5-point Likert scale: “This explanation includes a chart,” “... uses descriptive text,” “... provides numerical details,” “... is interactive,” and “... includes a population comparison”. For the manipulation check “... uses descriptive text” used for the textual condition, we acknowledge it may have been less discriminative because brief text was included in all conditions for minimal framing. We also included 2 attention checks, one early (in the Profile phase) and one late (in the Evaluation phase): “Please select ‘Completely wrong’ for this question to confirm that you are paying attention.”

### Data Analysis Strategy

To ensure data quality, we first retained only participants who passed both attention checks. To verify that each experimental manipulation worked as intended, we tested manipulation checks with separate linear models in which the dependent variable was endorsement of the corresponding item (Likert 1‐5; eg, “includes a chart”) and the independent variables were the explanation modalities. For each item, we set the targeted explanation modality as the reference (eg, the visual modality for “includes a chart”) and conducted 2-sided *t* tests comparing its endorsement with each other explanation modality.

To confirm that randomization produced comparable groups, we assessed sociodemographic balance across experimental conditions using Pearson chi-square tests for categorical variables (gender, children, income, and education) and a 2-sided Kruskal-Wallis test for age.

To test whether explanation modality related to explanation satisfaction, we used a linear model with explanation satisfaction as the dependent variable and the following independent variables: explanation modality (dummy-coded with no-explanation as the baseline), measured well-being score (total score of the 14-item MHC-SF), absolute prediction error (|predicted−measured| based on predictions generated by our model from 20 lifestyle items), and sociodemographic covariates (gender, born in Canada, having children, income, education, and age). We report unstandardized coefficients (B), SEs, and 2-sided *P* values.

Finally, to identify which explanation modalities differed from one another on explanation satisfaction, we conducted post hoc pairwise comparisons between explanation modalities based on the fitted model (implemented with emmeans) and controlled for multiple comparisons using Tukey-adjusted, 2-sided *t* tests. Across all analyses, statistical significance was evaluated at Cronbach α=0.05.

### Results

#### Participant Overview

A total of 1369 individuals were invited to take part in the study. Of these, 1276 (93.2%) completed the survey within the permitted duration, whereas 93 (6.8%) either discontinued the study before completion or timed out. Among the 1276 completed responses, 24 (1.9%) failed the attention checks and were removed. All subsequent analyses were conducted on the final sample of 1252 participants.

Overall, 660 out of 1252 (52.7%) identified as women, 541 out of 1252 (43.2%) as men, and 51 out of 1252 (4.1%) as another gender. Regarding education, 232 out of 1252 (18.5%) had a high school degree or less, 206 out of 1252 (16.5%) had completed college, and 814 out of 1252 (65%) had earned a university degree. A total of 372 out of 1252 (29.7%) reported having at least 1 child. Household income was distributed as ≤US $60,000 (376/1252, 30%), US $60,000-US $110,000 (452/1252, 36.1%), US $110,000-US $150,000 (209/1252, 16.7%), and ≥US $150,000 (216/1252, 17.3%). Participants’ age averaged 36.85 (SD 12.59) years, with a median of 34 (IQR 25-42) years and a range of 18‐81 years. Relative to the 2021 Canadian Census, the sample was younger and more highly educated, whereas gender distribution was similar. Income and parental status were not directly comparable.

Participants were randomly assigned to one of the following 6 experimental conditions: textual (n=215), interactive (n=212), quantitative (n=208), visual (n=208), baseline (n=205), and contextual (n=204).

Manipulation checks showed that the visual, quantitative, interactive, and contextual conditions received significantly higher endorsement on their corresponding manipulation-check item than the alternative conditions, using a significance threshold of *P*<.05, indicating successful manipulations. The textual condition showed significantly higher endorsement of “uses descriptive text” than the baseline, contextual, and interactive conditions, but not relative to the quantitative or visual conditions, consistent with the presence of brief textual content across explanation formats, which likely reduced the discriminative power of this manipulation check.

Sociodemographic characteristics did not differ significantly across the 6 experimental conditions. Gender distribution did not vary by condition (*χ*²_10_=9.8; *P*=.54). Similarly, education levels did not differ across conditions (*χ*²_10_=17.3; *P*=.07), nor did parental status (*χ*²_5_=4.4; *P*=.61) or household income (*χ*²_15_=15.4; *P*=.37). Age also did not differ significantly across conditions (Kruskal-Wallis H_5_=2.20; *P*=.79). Because randomization produced demographically comparable groups, sociodemographic variables were included as covariates in the main model.

To contextualize the use of prediction error as a covariate in study 2, we evaluated the predictive performance of the well-being model trained on study 1 when applied, without retraining, to the study 2 sample. On study 2 participants, the model achieved an MAE of 12.6 and an *R*² of 0.39, compared with an MAE of approximately 15.0 and an *R*² of approximately 0.28 in study 1 cross-validation. To further interpret this difference, we examined the distribution of the measured well-being outcome in both samples. In study 1 (n=1845), the rescaled well-being score had a mean of 57.31 (SD 21.39), whereas in study 2 (n=1249), the mean was 53.85 (SD 19.87) with a lower dispersion. This reduced outcome variance indicates a more homogeneous sample in study 2, consistent with its younger and more educated participant composition. Because both MAE and *R*² are sensitive to outcome dispersion, this narrower distribution likely contributes to the lower MAE and higher *R*² observed in study 2, without implying improved calibration or retraining of the model.

#### Model Results

Multimedia Appendix 8 reports full model estimates. Regarding explanation modality, all modalities showed a significant positive effect on explanation satisfaction compared with the baseline condition (no explanation): interactive (B=0.914, SE 0.076; *P*<.001), visual (B=0.915, SE 0.077; *P*<.001), textual (B=0.850, SE 0.076; *P*<.001), quantitative (B=0.782, SE 0.077; *P*<.001), and contextual (B=0.218, SE 0.077; *P*=.005).

The measured well-being score (total score of the 14-item MHC-SF) was positively associated with satisfaction (B=0.00691, SE 0.00116; *P*<.001), whereas the prediction error (|predicted−measured|) showed no association (B=−0.00341, SE 0.00240; *P*=.16).

Among sociodemographic covariates, having at least 1 child was positively associated with satisfaction (B=0.143, SE 0.050; *P*=.004). Significant positive associations were also observed between satisfaction and being a man (B=0.108, SE 0.045; *P*=.02) and between the US $60,000-US $110,000 income group versus ≤US $60,000 (B=0.140, SE 0.056; *P*=.01).

#### Post Hoc Comparisons

Post hoc comparisons indicated that the interactive, visual, textual, and quantitative explanation modalities yielded higher explanation satisfaction relative to the baseline condition (*P*<.001, Tukey-adjusted). The contextual modality showed a positive but nonsignificant difference after the Tukey adjustment (estimate 0.218, SE 0.077; *P*=.055, Tukey-adjusted). Among the nonbaseline conditions (those including an explanation), satisfaction in the contextual modality was significantly lower than satisfaction in the interactive, visual, textual, and quantitative modalities (*P*<.001, Tukey-adjusted). No significant differences were observed among these 4 modalities.

## Discussion

### Principal Findings

This research brought together 2 complementary components to examine how AI can support individuals’ understanding of their well-being. First, we developed an interpretable model grounded in key lifestyle and contextual factors; second, we evaluated how different explanation modalities shape users’ reactions to its feedback. These 2 strands provide the basis for the reflections that follow, beginning with the predictive insights from study 1 and then turning to the user-experience findings from study 2.

### Interpretation of Study 1 Findings

The predictive modeling conducted in study 1 explains approximately one-third of the variance in well-being (*R*²≈0.33). Given the multifactorial nature of well-being [129,130] where dispositional traits, proximal life circumstances, and more distal, structural conditions remain only partially observed, this represents a moderate yet meaningful level of explanatory power. MAE compares favorably to naïve baselines: the ridge model’s MAE (≈14.3) improves substantially over the mean regressor (≈17.8) and is far superior to a random regressor. In practical terms, predictions move beyond population averages to differentiate profiles reliably, enabling rank-ordering and targeted feedback while acknowledging residual uncertainty at the individual level.

The strongest positive coefficients concentrated on sleep quality, work autonomy (ability to decide when to take breaks), frequency of social activities with friends, satisfaction with neighborhood or social environment (friendliness and health-promoting opportunities), and volunteering. These align with established evidence linking restorative sleep to affect and life satisfaction [131]; job control to better outcomes [132]; social participation and social networks to better health [133-135]; place-based resources to healthier behaviors [136]; and prosocial engagement to purpose and eudaimonia [137]. Collectively, they foreground actionable levers that individuals and organizations can influence.

The most negative coefficients included indicators of limited workplace support (fewer opportunities to discuss difficulties), specific occupational domains associated with stressors or schedule irregularity, presence of chronic conditions, and recent mental-health consultations. These patterns are consistent with the literature, in which help-seeking and chronic illness mark elevated burden rather than causing lower well-being per se [138]. Occupational signals plausibly track exposure to physical or psychosocial risks and variability in autonomy [139,140]. Interpretation should remain nonstigmatizing: coefficient index association, not causality, and can guide supportive interventions (eg, improving supervisory support, facilitating accommodations, and strengthening access to preventive resources).

Taken together, study 1 indicates that a concise, low-redundancy questionnaire feeding a regularized linear model yields predictions that are accurate enough to inform meaningful, actionable feedback. Explanations should foreground modifiable behaviors and environmental contexts, while framing identified risk signals as opportunities for support and improvement rather than as person-level judgments. This foundation motivates the explanation-design choices evaluated in the subsequent stage.

### Interpretation of Study 2 Findings

Building on study 1’s model, study 2 aimed to examine how distinct explanation modalities affect users’ satisfaction with the model’s feedback, in the spirit of XAI.

A first finding from this study is that providing users with any form of explanation appears to be associated with greater user satisfaction. This result is consistent with long-standing theories in psychology and research in user experience, showing that people have a fundamental need to feel competent in their daily lives, including when interacting with an interface or technology. Competence is one of humans’ basic psychological needs and thus a core driver of behavior, cognition, and well-being [141]. Recent work in human-computer interaction has similarly demonstrated that technology interfaces that support users’ sense of competence are associated with higher levels of satisfaction and engagement [142]. By offering an explanation of how the AI model works, the system allows users to make sense of what would otherwise remain a “black box,” thereby strengthening their feeling of competence during the interaction.

A second finding from this study is that the contextual condition led to lower satisfaction compared to the other explanation modalities. In our experiment, the contextual condition focused on comparing a person’s well-being score with that of a reference group and did not provide any AI-derived information about the main factors they could act on to improve their well-being. This result is consistent with conceptual critiques of social comparison–based well-being or health apps, which emphasize that comparative feedback may have variable effects depending on the user and context. While some individuals may benefit from seeing how they compare to others, others may experience frustration or disengagement, particularly if such feedback is not accompanied by actionable guidance or tailored to their preferences and comparison orientation [124,143]. This is also aligned with AI research showing that AI-based interventions tend to be more effective when algorithmic transparency is higher [22,25,144].

A third finding is that comparisons among the explanation modalities (other than the contextual ones) did not reveal any statistically significant differences in their effects on user satisfaction. Nevertheless, the visual and interactive modalities showed somewhat stronger associations with satisfaction, suggesting that they may exert slightly larger effects than quantitative or purely textual explanations. This pattern supports the idea that, beyond simply providing *any* form of model-based explanation, user satisfaction can be modestly improved by adopting more engaging, easy-to-interpret, and visually appealing forms of explanation. Interactivity can enhance users’ understanding of the model, strengthen their sense of control, and reduce both cognitive load and anxiety [145]. Visualization also has the potential to reduce users’ cognitive load and help increase their confidence [146].

Finally, a last finding is that individuals with higher well-being levels reported greater satisfaction with the explanations provided, regardless of the explanation modality. This result aligns with well-being research highlighting that positive emotions, which are key components of well-being, can promote exploration in life [147], are associated with more acceptance of technologies [148], and are key to health interventions’ effects [149]. In contrast, individuals with lower well-being levels are more likely to experience negative affect, such as nervousness or frustration, which could act as barriers to their engagement with health interventions [150].

### Theoretical and Practical Cross-Study Implications

This research extends XAI into the underexplored domain of general psychological well-being. We show that well-being can be predicted meaningfully using a small set of relevant and actionable predictors, supporting the idea that it can be modeled in a parsimonious and practical way. Our results further indicate that different XAI modalities vary in the degree to which they are perceived as satisfying by users when interpreting model outputs. Visual and interactive formats, in particular, were associated with higher explanation satisfaction and may represent promising approaches for supporting users’ engagement with and perceived understanding of well-being–related feedback. However, outcomes such as actual comprehension and behavior change were not assessed in this study.

A key insight concerns the dynamic between model and user: interpretable models, such as ridge regression, appear to foster interpretable user experiences. Simpler, more transparent approaches enable users to build coherent, credible, and personally meaningful explanations. We also find that baseline well-being levels shape satisfaction across XAI modalities. Low well-being may hinder engagement with AI-based mobile apps—even those not focused on mental health—highlighting the need to understand how emotional states create barriers and how XAI can reduce them.

Our study is among the first randomized controlled experiments in well-being to offer empirical guidance on which explanation modalities people find most satisfying. Such evidence is urgently needed as mobile apps increasingly embed AI to support self-care, yet rarely disclose empirical validation of their XAI practices. The scarcity of rigorous research, including randomized designs, suggests many existing approaches lack an evidence base and may yield suboptimal user outcomes.

Based on our findings, we recommend that developers of AI-supported well-being systems prioritize visual and interactive explanations over abstract narrative, quantitative, or socially comparative formats. Clarity and transparency should take precedence over mathematical performance or technical sophistication alone.

### Limitations and Future Work

Across both studies, several limitations warrant consideration. Both relied on online, self-selected samples. Study 1 used the Léger panel, which follows census-aligned quotas but may overrepresent experienced survey takers. Study 2 recruited from Prolific, where participants are typically younger, more educated, and digitally skilled. Although sociodemographic controls were applied, these platform-specific biases may limit generalizability, underscoring the need for replication in more diverse or probability-based samples.

In study 1, all predictors and the well-being outcome were self-reported, making results susceptible to recall bias, momentary affect, and social desirability. Despite validated instruments and careful item selection, common-method bias cannot be excluded. Moreover, the cross-sectional design identifies correlations rather than causal relationships between behaviors and well-being; longitudinal or experimental work is needed to test whether behavioral changes drive genuine improvements.

The model’s moderate predictive accuracy (*R*²≈0.28 in study 1 and *R*²≈0.39 in study 2) and reliance on regularized linear regression reflect both interpretability goals and practical constraints, including sample size and measurement noise. While enhancing transparency, this choice limits the detection of complex, nonlinear dynamics underlying well-being.

Study 2 adds further constraints. Its web-based setting may have introduced variability linked to digital literacy, device type, or comfort with interactive tools. The single-session format also limits ecological validity, as real-world well-being tools typically involve repeated engagement. Furthermore, although explanations improved satisfaction, the present design does not allow conclusions regarding users’ actual comprehension or subsequent behavioral change. Nevertheless, lower satisfaction reported among participants with lower well-being suggests that explanation strategies may need to adapt to users’ emotional and cognitive states.

Future research should validate these findings in larger and more heterogeneous datasets, ideally integrating objective behavioral indicators such as sleep or activity data to reduce reliance on self-report. As richer data become available, hybrid and nonlinear models could be explored while preserving interpretability through tailored explanation interfaces. Explanations themselves should evolve toward personalization, adjusting content, format, and complexity to user profiles and well-being levels.

Additionally, it is important to clarify the status of causal interpretation in this work. Although causality is a central concern in the design of interventions for well-being, the predictive models used here are trained on observational, cross-sectional data and therefore capture statistical associations rather than causal relationships. Accordingly, feature-based attributions should be interpreted as illustrating how the model’s predictions would change under hypothetical input modifications, not as claims that such changes would necessarily cause improvements in well-being in the real-world. Establishing causal effects in this domain would require stronger assumptions, longitudinal or experimental designs, and explicit causal modeling. Extending evaluation beyond 1-time interactions is crucial. Testing explanations in sustained, real-world contexts, such as mobile or digital well-being tools, would help determine whether higher explanation satisfaction is associated with longer-term outcomes, including sustained engagement, improved understanding, or behavioral change. Embedding explanations within adaptive feedback loops could transform XAI systems from static information displays into dynamic, supportive companions for well-being.

Finally, our feature-based explanation modalities in study 2 (ie, quantitative, textual, visual, and interactive) deliberately focused only on features with the largest positive coefficients in the regression model. Although this design choice aimed to keep explanations consistent across modalities and strengths-oriented in a sensitive domain (Multimedia Appendix 7), it provides only a partial view of the model’s reasoning. Future work could also incorporate features with the largest negative coefficients and assess how presenting both positive and negative features affects user satisfaction.

### Conclusion

This work demonstrates that explainable AI can foster more satisfying user experiences of interactions with an AI system about their well-being when models are designed and communicated with end users in mind. Across 2 complementary studies, we show that an interpretable model can capture a substantial portion of variance in subjective well-being using a concise set of actionable lifestyle factors. Visual and interactive explanations consistently fostered the greatest satisfaction. By contrast, population-comparison feedback offered limited value.

As AI becomes increasingly embedded in personal health and self-care technologies, these insights offer concrete, evidence-based design principles. Systems should prioritize clarity over technical novelty, emphasize actionable behavioral pathways over social comparisons, and use visual or interactive formats that create a sense of competence and control. Looking ahead, longer-term and ecologically grounded studies are needed to determine how explanations shape long-term engagement, trust calibration, and real behavioral change. Integrating multimodal data streams, adaptive personalization, and iterative feedback loops may further enhance both predictive accuracy and user empowerment.

Overall, this research provides a foundation for developing AI tools that are not only accurate but also understandable, humane, and supportive of people’s everyday efforts to care for their well-being.

## Supplementary material

10.2196/88750 Multimedia Appendix 1 Questionnaire study 1.

10.2196/88750 Multimedia Appendix 2 Technical details for the machine learning pipeline of study 1.

10.2196/88750 Multimedia Appendix 3 Variance inflation factor analysis.

10.2196/88750 Multimedia Appendix 4 Ridge regression coefficients.

10.2196/88750 Multimedia Appendix 5 Questionnaire study 2.

10.2196/88750 Multimedia Appendix 6 Interfaces for the 6 explanation modalities.

10.2196/88750 Multimedia Appendix 7 Technical details for explanation modalities design.

10.2196/88750 Multimedia Appendix 8 Explanation satisfaction model results.

## Abbreviations

AI: artificial intelligence
LLM: large language model
MAE: mean absolute error
MHC-SF: Mental Health Continuum-Short Form
ML: machine learning
MSE: mean square error
XAI: explainable artificial intelligence
XGBoost: extreme gradient boosting
