# Supplementary material for: Explainable AI for Well-Being Prediction From Lifestyle Data: 2-Study Design
Source: JMIR Ment Health. 2026 May 8;13:e88750. doi: 10.2196/88750 (PMC13155431; doi:10.2196/88750)
Supplement: Multimedia Appendix 1 [file mental-v13-e88750-s001.pdf]

Questionnaire – Study 1

Source information The "Source" indicates the reference of the scale or instrument that informed each item, whether the item was inspired by that measure, adapted from it, or uses the same wording. Sources are provided only for items originating from validated instruments.

Note: Questions marked with a star (*) have been selected during the feature selection phase and are used in the prediction model for Study 2.

Question 1

Question id : `genre`

Type of variable : nominal_single

Question content : The next questions will focus on your demographic characteristics. More generally, these questions will help us build your profile.

What is your gender?

Possible answers : - Male (cisgender male): 1 - Female (cisgender female): 2 - Male (transgender male): 3 - Female (transgender woman): 4 - Non-binary: 5 - Queer: 6 - Agender: 7 - Other: 8

Question 2

Question id : `orientation`

Type of variable : nominal_single

Question content : What is your sexual orientation?

Possible answers : - Heterosexual: 1 - Bisexual: 2 - Gay or lesbian: 3 - Other: 4

Question 3

Question id : `age`

Type of variable : integer

Question content : How old are you? Please indicate your age using a number.

Possible answers : - 18: 18 - 19: 19 - 20: 20 - 21: 21 - 22: 22 - 23: 23 - 24: 24 - 25: 25 - 26: 26 - 27: 27 - 28: 28 - 29: 29 - 30: 30 - 31: 31 - 32: 32 - 33: 33 - 34: 34 - 35: 35 - 36: 36 - 37: 37 - 38: 38 - 39: 39 - 40: 40 - 41: 41 - 42: 42 - 43: 43 -

44: 44 - 45: 45 - 46: 46 - 47: 47 - 48: 48 - 49: 49 - 50: 50 - 51: 51 - 52: 52 - 53: 53 - 54: 54 - 55: 55 - 56: 56 - 57: 57 - 58: 58 - 59: 59 - 60: 60 - 61: 61 - 62: 62 - 63: 63 - 64: 64 - 65: 65 - 66: 66 - 67: 67 - 68: 68 - 69: 69 - 70: 70 - 71: 71 - 72: 72 - 73: 73 - 74: 74 - 75: 75 - 76: 76 - 77: 77 - 78: 78 - 79: 79 - 80: 80 - 81: 81 - 82: 82 - 83: 83 - 84: 84 - 85: 85 - 86: 86 - 87: 87 - 88: 88 - 89: 89 - 90: 90 - 91: 91 - 92: 92 - 93: 93 - 94: 94 - 95: 95 - 96: 96 - 97: 97 - 98: 98 - 99: 99

Question 4

Question id : `langue_maternelle`

Type of variable : nominal_single

Question content : What language do you speak most often at home?

Possible answers : - French: 1 - English: 2 - Other: 3

Question 5

Question id : `occupation_1`

Type of variable : nominal_multiple

Question content : What is your primary occupation? - Selected Choice

Possible answers : - Self-employed (with or without employees): 1 - Salaried work (full-time, part-time, paid leave): 2 - Retired: 3 - Unemployed, looking for work: 4 - Student: 5 - Caregiver: 6 - Parent at home: 7 - Unemployed due to a handicap or disability: 8 - Multiple paid jobs (self-employment): 9 - Multiple paid jobs (salaried work): 12 - Unemployed, receiving social assistance or social solidarity benefits: 13 - Other (specify): 10

Question 6 (*)

Question id : `travail_domaine_1`

Type of variable : nominal_multiple

Question content : Which one of the following categories best describes your field of employment? - Selected Choice

Possible answers : - Management: 1 - Business, finance and administration: 2 - Natural and applied sciences and related fields: 3 - Health: 4 - Education, law and social, community and government services: 5 - Arts, Culture, Sport and Recreation: 6 - Sales and Service: 7 - Trades, transport, equipment operators and

related occupations: 8 - Natural Resources, Agriculture and Related Production: 9 - Manufacturing and utilities: 10 - Other (specify): 11

Question 7

Question id : `travail_heures`

Type of variable : float

Question content : How many hours on average do you work per week? Please indicate the number of hours using a figure.

Question 8

Question id : `teletravail_heures`

Type of variable : float

Question content : Of these hours, approximately how many do you spend working from home (teleworking)? Please indicate the number of hours using a figure.

Question 9

Question id : `revenu`

Type of variable : ordinal

Question content : Approximately, which of the following categories does your total household income, before taxes, fall into?

Possible answers : - No income: 1 - $1 to $30,000: 2 - $30,001 to $60,000: 3 - $60,001 to $90,000: 4 - $90,001 to $110,000: 5 - $110,001 to $150,000: 6 - $150,001 to $200,000: 7 - More than $200,000: 8

Question 10

Question id : `education`

Type of variable : ordinal

Question content : What is the highest level of education that you have completed?

Possible answers : - No schooling: 1 - Elementary school: 2 - High school: 3 - College, CEGEP, or Classical College: 4 - Bachelor's degree: 5 - Master's degree: 6 - PhD: 7

Question 11 (*)

Question id : `origines_ethniques`

Type of variable : nominal_single

Question content : Which of the following categories best describes you?

Possible answers : - White: 1 - Black: 2 - Indigenous: 3 - Asian: 4 - Hispanic: 5 - Arab: 6 - Other: 7

Question 12

Question id : `religiosite_1`

Type of variable : integer

Question content : On a scale of 0 to 100, how important is religion in your life? - Importance of religion

---

Question 13

Question id : `enfants`

Type of variable : ordinal

Question content : How many children under the age of 18 live with you?

Possible answers : - 0: 1 - 1: 2 - 2: 3 - 3: 4 - 4: 5 - 5 or more: 6

Question 14 (*)

Question id : `married`

Type of variable : nominal_single

Question content : What is your marital status?

Possible answers : - Single: 1 - Married: 2 - Common-law relationship: 3 - Widower/widow: 4 - Divorced/separated: 5

Question 15

Question id : `travail_deplacement`

Type of variable : ordinal

Question content : How long does it usually take you to get from your home to your workplace, according to your usual mode of transport?

Possible answers : - Less than 15 minutes: 1 - Between 15 and 30 minutes: 2 - Between 30 minutes and an hour: 3 - Between 1 and 2 hours: 4 - More than 2 hours: 5 - I don't work outside the home: 6

Question 16

Question id : `milieu_vie`

Type of variable : nominal_single

Question content : How would you describe the place where you live?

Possible answers : - City: 1 - Suburb: 2 - Small town: 3 - Rural area/village: 4

Question 17

Question id : `habitation`

Type of variable : nominal_single

Question content : Which of the following categories best describes your type of dwelling? - Selected Choice

Possible answers : - Apartment in a building that has fewer than five storeys: 1 - Loft: 2 - Condo: 3 - High-rise apartment building: 4 - Detached house: 5 - Townhouse: 6 - Duplex: 7 - Cooperative: 8 - Social housing: 9 - Mobile home (boat, van, RV, etc.): 10 - Other (please specify): 11

Question 18

Question id : `pets`

Type of variable : nominal_single

Question content : We're now going to ask you some questions about your lifestyle. More generally, these questions will help us gain a better understanding of your daily life.

Do you have pets?

Possible answers : - Cat(s): 1 - Dogs): 2 - Cat(s) and dog(s): 3 - Other Pet(s): 4 - Farm animals: 5 - I don't have pets: 6

Question 19

Question id : `act_physique`

Type of variable : nominal_single

Question content : What physical activity do you do most often?

Possible answers : - Run: 1 - Gym: 2 - Walk: 3 - Swim: 4 - Team sport: 5 - Yoga: 6 - Other: 7 - I don't do physical activities: 8

Question 20

Question id : `freq_physique_`

Type of variable : ordinal

Question content : How often are you physically active?

Possible answers : - Never: 1 - Sometimes: 2 - Often: 3 - Very often: 4

Question 21

Question id : `transport`

Type of variable : nominal_single

Question content : What is your main mean of transportation?

Possible answers : - Public transit: 1 - Walking: 2 - Motorbike: 3 - Motorcycle: 4 - Car: 5

Question 22 (*)

Question id : `car_model`

Type of variable : nominal_single

Question content : Which of the following car models do you happen to use most often?

Possible answers : - 4x4: 1 - Regular sedan or station wagon: 2 - Convertible or roadster: 3 - Pickup: 4 - Van or minivan: 5 - Luxury car (Mercedes, Porsche, etc.): 6 - Sports car: 7 - Hybrid or electric car: 8 - SUV: 9 - Other: 10 - I do not have a car or I never use a car: 11

Question 23

Question id : `consumption`

Type of variable : nominal_single

Question content : Where do you usually buy your clothes?

Possible answers : - Independent stores: 1 - Chain stores (Gap, Zara, etc.): 2 - Thrift stores: 3 - Superstores (Walmart, Costco, etc.): 4 - Department stores (The Bay, Simons, etc.): 5 - Online only stores: 6 - Other: 7

Question 24

Question id : `coffee`

Type of variable : nominal_single

Question content : Where do you usually get your coffee?

Possible answers : - Independent coffee shops: 1 - McDonald's: 2 - Second Cup: 3 - Starbucks: 4 - Tim Hortons: 5 - Other coffee shop chains: 6 - I don't go to coffee shops: 7

Question 25

Question id : `meat`

Type of variable : ordinal

Question content : Do you eat meat?

Possible answers : - Never: 1 - A few times a year: 2 - Once a month: 3 - Once a week: 4 - A few times a week: 5 - Once a day: 6 - More than once a day: 7

Question 26

Question id : `alcool_pref`

Type of variable : nominal_single

Question content : What is your favourite type of alcohol?

Possible answers : - Craft or microbrewery beer: 1 - Regular beer: 2 - Spirit drink: 3 - Cocktail: 4 - White wine: 5 - Sparkling wine or champagne: 6 - Rosé wine: 7 - Red wine: 8 - I do not drink alcohol: 9

Question 27 (*)

Question id : `smoking`

Type of variable : ordinal

Question content : How often do you smoke cigarettes and/or vape?

Possible answers : - Never: 1 - A few times a year: 2 - Once a month: 3 - Once a week: 4 - A few times a week: 5 - Once a day: 6 - More than once a day: 7

Question 28

Question id : `cannabis`

Type of variable : ordinal

Question content : How often do you use cannabis (pot, marijuana, hashish)?

Possible answers : - Never: 1 - A few times a year: 2 - Once a month: 3 - Once a week: 4 - A few times a week: 5 - Once a day: 6 - More than once a day: 7

Question 29

Question id : `act_hunting`

Type of variable : ordinal

Question content : How often do you hunt?

Possible answers : - Never: 1 - Almost never: 2 - Sometimes: 3 - Often: 4 - Very often: 5

Question 30

Question id : `act_fishing`

Type of variable : ordinal

Question content : How often do you fish?

Possible answers : - Never: 1 - Almost never: 2 - Sometimes: 3 - Often: 4 - Very often: 5

Question 31

Question id : `act_motorized`

Type of variable : ordinal

Question content : How often do you do motorized outdoor activities (jet ski, snowmobile, etc.)?

Possible answers : - Never: 1 - Almost never: 2 - Sometimes: 3 - Often: 4 - Very often: 5

Question 32 (*)

Question id : `act_friends`

Type of variable : ordinal

Question content : How often do you do activities with one or more friend(s)?

Possible answers : - Never: 1 - Almost never: 2 - Sometimes: 3 - Often: 4 - Very often: 5

Question 33 (*)

Question id : `act_volunteer`

Type of variable : ordinal

Question content : How often do you volunteer or involve yourself in a cause?

Possible answers : - Never: 1 - Almost never: 2 - Sometimes: 3 - Often: 4 - Very often: 5

Question 34

Question id : `act_museum`

Type of variable : ordinal

Question content : How often do you visit museums and/or art galleries?

Possible answers : - Never: 1 - Almost never: 2 - Sometimes: 3 - Often: 4 - Very often: 5

Question 35 (*)

Question id : `act_nature_1`

Type of variable : ordinal

Question content : How often do you spend time in green or natural environments? - May to September

Possible answers : - Never: 1 - Almost never: 2 - Sometimes: 3 - Often: 4 - Very often: 5

Question 36

Question id : `act_nature_2`

Type of variable : ordinal

Question content : How often do you spend time in green or natural environments? - October to April

Possible answers : - Never: 1 - Almost never: 2 - Sometimes: 3 - Often: 4 - Very often: 5

Question 37 (*)

Question id : `style`

Type of variable : nominal_single

Question content : What is your clothing style? - Selected Choice

Possible answers : - Hippie: 1 - Elegant: 2 - Classical: 3 - Casual: 4 - Formal: 5 - Punk: 6 - Rock: 7 - Sporty: 8 - Other (please specify): 9

Question 38 (*)

Question id : `maladies_1`

Type of variable : nominal_multiple

Question content : The following questions are about "long-term health conditions" which are expected to last or have already lasted 6 months or more and that have been diagnosed by a health professional. Chronic health problems (e.g. cancer, hypertension, cardiovascular disease, diabetes, asthma, chronic obstructive pulmonary disease, arthritis, intestinal disease, etc.)

Source : Statistics Canada. Canadian Community Health Survey (CCHS) - Mental Health. 2015. Available from:

Url link : Open link

Possible answers : Chronic health problems (e.g. cancer, hypertension, cardiovascular disease, diabetes, asthma, chronic obstructive pulmonary disease, arthritis, intestinal disease, etc.): 1

Question 39 (*)

Question id : `autogestion_9`

Type of variable : ordinal

Question content : We're now going to ask you some questions about your health behaviours and psychological well-being. More generally, these questions will help us gain a better understanding of your physical and mental health.

Here is a list of ways you may be using to feel well or better, to maintain good mental health or avoid psychological difficulties. For each tool, indicate how often you have used it in the past month. - I have a healthy diet.

Source : Coulombe, S., Radziszewski, S., Trépanier, S. G., Provencher, H., Roberge, P., Hudon, C., … & Houle, J. (2015). Mental health self-management questionnaire: Development and psychometric properties. Journal of Affective Disorders, 181, 41-49.

Possible answers : - Never: 1 - Rarely: 2 - Often: 3 - Very often: 4

Question 40

Question id : `autogestion_10`

Type of variable : ordinal

Question content : We're now going to ask you some questions about your health behaviours and psychological well-being. More generally, these questions will help us gain a better understanding of your physical and mental health.

Here is a list of ways you may be using to feel well or better, to maintain good mental health or avoid psychological difficulties. For each tool, indicate how often you have used it in the past month. - I do exercises to relax (yoga, tai chi, breathing techniques, etc.).

Source : Coulombe S, Radziszewski S, Trépanier S-G, Provencher H, Roberge P, Hudon C, Meunier S, Provencher MD, Houle J. Mental health self-management questionnaire: Development and psychometric properties. Journal of Affective Disorders 2015 Aug 1;181:41–49. doi: 10.1016/j.jad.2015.04.007

Possible answers : - Never: 1 - Rarely: 2 - Often: 3 - Very often: 4

Question 41 (*)

Question id : `sommeil_1`

Type of variable : float

Question content : The following question refers to your overall sleep quality for the majority of nights in the past seven days only. Please think about the quality of your sleep overall, such as how many hours of sleep you got, how easily you fell asleep, how often you woke up during the night (except to go to the bathroom), how often you woke up earlier than you had to in the morning, and how refreshing your sleep was. During the past seven days, how would you rate your sleep quality overall? - Sleep quality

Source : Snyder E, Cai B, DeMuro C, Morrison MF, Ball W. A new single-item sleep quality scale: results of psychometric evaluation in patients with chronic primary insomnia and depression. Journal of Clinical Sleep Medicine American Academy of Sleep Medicine; 2018;14(11):1849–1857. doi: 10.5664/jcsm.7478

Question 42 (*)

Question id : `chronotype`

Type of variable : nominal_single

Question content : Self-assess your own chronotype by choosing a graph representing the evolution of your level of alertness over the course of the day.

Source : Putilov AA, Sveshnikov DS, Puchkova AN, Dorokhov VB, Bakaeva ZB, Yakunina EB, . . . , Mairesse O. Single-Item Chronotyping (SIC), a method

to self-assess diurnal types by using 6 simple charts. Personality and Individual Differences 2021 Jan 1;168:110353. doi: 10.1016/j.paid.2020.110353

Possible answers : - Morning: 1 - Evening: 2 - Highly active: 3 - Daytime sleep: 4 - Diurnal: 5 - Moderately active: 6

Question 43

Question id : `alim_fruits__leg`

Type of variable : ordinal

Question content : Generally speaking, how many servings of fruit and vegetables do you eat on average each day (total fruit and vegetable servings)? Definition of a portion of fruit or vegetables: A portion of fruit or vegetables includes : - one medium-sized fruit or vegetable (e.g.: 1 carrot, 1 tomato, 1 apple, 1 pear); - half a cup (125 ml) of chopped berries or vegetables (equivalent to the size of a tennis ball); - half a cup (125 ml) of 100% pure juice with no added sugar; -250 ml (1 cup) of leafy vegetables or salad; - 60 ml (1/4 cup) of dried fruit (e.g.: grapes, cranberries, apricots). Food may be fresh, frozen or canned.

Source : Panahi S, Frappier I, Gagnon J, Sørensen CV, Jacob R, Tremblay A, Drapeau V. A brief dietary screener to assess diet quality and lifestyle factors in a university community. Unpublished document. 2025.

Possible answers : - Less than 1 portion per day: 1 - 1 portion per day: 2 - 2 portion per day: 3 - 3 portion per day: 4 - 4 portion per day: 5 - 5 portion per day: 6 - 6 portion per day: 7 - 7 or more portion per day: 8 - I don't consume any: 9

Question 44

Question id : `conso_freq`

Type of variable : ordinal

Question content : Over the past 12 months, how often have you consumed alcohol?

Source : Tremblay J, Blanchette-Martin N, Ferland F, Goyette M, Dufour M, Bertrand K, Ménard JM, L'Espérance N, Brochu S, Giroux I, Savard AC, Rousseau M, Landry M, Bergeron J, Turcotte S. Développement et validation d'une évaluation intégrée spécialisée en dépendance (ÉISD). Recherche et intervention sur les substances psychoactives – Québec (RISQ); 2019.

Possible answers : - Never: 1 - Once: 2 - A few times: 3 - Every month: 4 - 1 to 2 times a week: 5 - Several times a week: 6 - Every day: 7

Question 45

Question id : `conso_drogue__1`

Type of variable : ordinal

Question content : In the last 12 months, how often have you : - used cannabis (marijuana, hashish, oil, wax, cannabinoids synth.)?

Source : Tremblay J, Blanchette-Martin N, Ferland F, Goyette M, Dufour M, Bertrand K, Ménard JM, L'Espérance N, Brochu S, Giroux I, Savard AC, Rousseau M, Landry M, Bergeron J, Turcotte S. Développement et validation d'une évaluation intégrée spécialisée en dépendance (ÉISD). Recherche et intervention sur les substances psychoactives – Québec (RISQ); 2019.

Possible answers : - Never: 1 - Once: 2 - A few times: 3 - Every month: 4 - 1 to 2 times a week: 5 - Several times a week: 6 - Every day: 7

Question 46

Question id : `conso_drogue__2`

Type of variable : ordinal

Question content : In the last 12 months, how often have you : - used amphetamines (methamphetamines, speeds, Vyvanse®, Concerta®, Ritalin®)?

Source : Tremblay J, Blanchette-Martin N, Ferland F, Goyette M, Dufour M, Bertrand K, Ménard JM, L'Espérance N, Brochu S, Giroux I, Savard AC, Rousseau M, Landry M, Bergeron J, Turcotte S. Développement et validation d'une évaluation intégrée spécialisée en dépendance (ÉISD). Recherche et intervention sur les substances psychoactives – Québec (RISQ); 2019.

Possible answers : - Never: 1 - Once: 2 - A few times: 3 - Every month: 4 - 1 to 2 times a week: 5 - Several times a week: 6 - Every day: 7

Question 47

Question id : `conso_drogue__3`

Type of variable : ordinal

Question content : In the last 12 months, how often have you : - used tobacco (e.g. cigarettes, cigars, pipe, vaporizer)?

Source : Tremblay J, Blanchette-Martin N, Ferland F, Goyette M, Dufour M, Bertrand K, Ménard JM, L'Espérance N, Brochu S, Giroux I, Savard AC,

Rousseau M, Landry M, Bergeron J, Turcotte S. Développement et validation d'une évaluation intégrée spécialisée en dépendance (ÉISD). Recherche et intervention sur les substances psychoactives – Québec (RISQ); 2019.

Possible answers : - Never: 1 - Once: 2 - A few times: 3 - Every month: 4 - 1 to 2 times a week: 5 - Several times a week: 6 - Every day: 7

Question 48

Question id : `DemPsy_1`

Type of variable : ordinal

Question content : We're now going to ask you some questions about your living environment. More generally, these questions will help us gain a better understanding of your working environment.

Please indicate the extent to which you agree with the following statements. - At work I often lack the time to complete my tasks.

Source : Gilbert-Ouimet M, Truchon M, Aubé K. Validation of the French version of the Questionnaire on Psychosocial Risks, Well-being, and Health at Work (QRBEST). Unpublished document. 2025.

Possible answers : - Strongly disagree: 1 - Disagree: 2 - More or less in agreement: 3 - Agree: 4 - Strongly agree: 5

Question 49

Question id : `DemPsy_5`

Type of variable : ordinal

Question content : We're now going to ask you some questions about your living environment. More generally, these questions will help us gain a better understanding of your working environment.

Please indicate the extent to which you agree with the following statements. - At work I am frequently interrupted or disturbed.

Source : Gilbert-Ouimet M, Truchon M, Aubé K. Validation of the French version of the Questionnaire on Psychosocial Risks, Well-being, and Health at Work (QRBEST). Unpublished document. 2025.

Possible answers : - Strongly disagree: 1 - Disagree: 2 - More or less in agreement: 3 - Agree: 4 - Strongly agree: 5

Question 50

Question id : `DemPsy_7`

Type of variable : ordinal

Question content : We're now going to ask you some questions about your living environment. More generally, these questions will help us gain a better understanding of your working environment.

Please indicate the extent to which you agree with the following statements. - At work I'm often forced to work overtime.

Source : Gilbert-Ouimet M, Truchon M, Aubé K. Validation of the French version of the Questionnaire on Psychosocial Risks, Well-being, and Health at Work (QRBEST). Unpublished document. 2025.

Possible answers : - Strongly disagree: 1 - Disagree: 2 - More or less in agreement: 3 - Agree: 4 - Strongly agree: 5

Question 51

Question id : `DemPsy_9`

Type of variable : ordinal

Question content : We're now going to ask you some questions about your living environment. More generally, these questions will help us gain a better understanding of your working environment.

Please indicate the extent to which you agree with the following statements. - My job requires physical effort.

Source : Gilbert-Ouimet M, Truchon M, Aubé K. Validation of the French version of the Questionnaire on Psychosocial Risks, Well-being, and Health at Work (QRBEST). Unpublished document. 2025.

Possible answers : - Strongly disagree: 1 - Disagree: 2 - More or less in agreement: 3 - Agree: 4 - Strongly agree: 5

Question 52 (*)

Question id : `LatDec_3`

Type of variable : ordinal

Question content : In the same context, please respond to the following statements. - I can decide when to take a break.

Source : Gilbert-Ouimet M, Truchon M, Aubé K. Validation of the French version of the Questionnaire on Psychosocial Risks, Well-being, and Health at Work (QRBEST). Unpublished document. 2025.

Possible answers : - Strongly agree: 1 - Agree: 2 - More or less in agreement: 3 - Disagree: 4 - Strongly disagree: 5

Question 53 (*)

Question id : `SoutSup_6`

Type of variable : ordinal

Question content : Please answer the following statements by selecting the one that applies to you. - There are times to discuss the difficulties involved in carrying out our task.

Source : Gilbert-Ouimet M, Truchon M, Aubé K. Validation of the French version of the Questionnaire on Psychosocial Risks, Well-being, and Health at Work (QRBEST). Unpublished document. 2025.

Possible answers : - Strongly agree: 1 - Agree: 2 - More or less in agreement: 3 - Disagree: 4 - Strongly disagree: 5

Question 54

Question id : `quartier_domicile_1`

Type of variable : ordinal

Question content : We're now going to ask you some questions about your living environment. More generally, these questions will help us gain a better understanding of your neighborhood.

We would like to know your perception of the neighborhood where your home is located. The neighborhood refers to the area within approximately a 10-15 minute walk from home. Using the following scale, please indicate your level of agreement with each of the statements. - Bike paths and pedestrian lanes allow safe circulation in the neighborhood.

Source : Coulombe, S., Meunier, S., Cloutier, L., Auger, N., Roy, B., Tremblay, G., ... & Houle, J. (2017). Health-promoting home and workplace neighborhoods: associations with multiple facets of men's health. American Journal of Men's Health, 11(6), 1680-1691.

Possible answers : - Strongly disagree: 1 - Somewhat disagree: 2 - Somewhat agree: 3 - Strongly agree: 4

Question 55

Question id : `quartier_domicile_2`

Type of variable : ordinal

Question content : We're now going to ask you some questions about your living environment. More generally, these questions will help us gain a better understanding of your neighborhood.

We would like to know your perception of the neighborhood where your home is located. The neighborhood refers to the area within approximately a 10-15 minute walk from home. Using the following scale, please indicate your level of agreement with each of the statements. - Fresh fruits and vegetables are accessible within walking distance in the neighborhood.

Source : Coulombe S, Meunier S, Cloutier L, Auger N, Roy B, Tremblay G, de Montigny F, Gaboury I, Bernard F-O, Lavoie B, Dion H, Houle J. Health-promoting home and workplace neighborhoods: associations with multiple facets of men's health. Am J Mens Health 2017 Nov;11(6):1680–1691. PMID:29073845

Possible answers : - Strongly disagree: 1 - Somewhat disagree: 2 - Somewhat agree: 3 - Strongly agree: 4

Question 56 (*)

Question id : `quartier_domicile_3`

Type of variable : ordinal

Question content : We're now going to ask you some questions about your living environment. More generally, these questions will help us gain a better understanding of your neighborhood.

We would like to know your perception of the neighborhood where your home is located. The neighborhood refers to the area within approximately a 10-15 minute walk from home. Using the following scale, please indicate your level of agreement with each of the statements. - The neighborhood's population is friendly (e.g., people smile or greet when they meet, help each other, etc.).

Source : Coulombe S, Meunier S, Cloutier L, Auger N, Roy B, Tremblay G, de Montigny F, Gaboury I, Bernard F-O, Lavoie B, Dion H, Houle J. Health-promoting home and workplace neighborhoods: associations with multiple facets of men's health. Am J Mens Health 2017 Nov;11(6):1680–1691. PMID:29073845

Possible answers : - Strongly disagree: 1 - Somewhat disagree: 2 - Somewhat agree: 3 - Strongly agree: 4

Question 57

Question id : `quartier_domicile_4`

Type of variable : ordinal

Question content : We're now going to ask you some questions about your living environment. More generally, these questions will help us gain a better understanding of your neighborhood.

We would like to know your perception of the neighborhood where your home is located. The neighborhood refers to the area within approximately a 10-15 minute walk from home. Using the following scale, please indicate your level of agreement with each of the statements. - The neighborhood is well-maintained and looks nice (e.g., cleanliness, presence of trees, interesting buildings, or attractive shops, etc.).

Source : Coulombe S, Meunier S, Cloutier L, Auger N, Roy B, Tremblay G, de Montigny F, Gaboury I, Bernard F-O, Lavoie B, Dion H, Houle J. Health-promoting home and workplace neighborhoods: associations with multiple facets of men's health. Am J Mens Health 2017 Nov;11(6):1680–1691. PMID:29073845

Possible answers : - Strongly disagree: 1 - Somewhat disagree: 2 - Somewhat agree: 3 - Strongly agree: 4

Question 58

Question id : `quartier_domicile_5`

Type of variable : ordinal

Question content : We're now going to ask you some questions about your living environment. More generally, these questions will help us gain a better understanding of your neighborhood.

We would like to know your perception of the neighborhood where your home is located. The neighborhood refers to the area within approximately a 10-15 minute walk from home. Using the following scale, please indicate your level of agreement with each of the statements. - Sports facilities are accessible in the neighborhood (e.g., swimming pools, fitness centers, soccer or tennis courts).

Source : Coulombe S, Meunier S, Cloutier L, Auger N, Roy B, Tremblay G, de Montigny F, Gaboury I, Bernard F-O, Lavoie B, Dion H, Houle J. Health-promoting home and workplace neighborhoods: associations with multiple facets of men's health. Am J Mens Health 2017 Nov;11(6):1680–1691. PMID:29073845

Possible answers : - Strongly disagree: 1 - Somewhat disagree: 2 - Somewhat agree: 3 - Strongly agree: 4

Question 59

Question id : `quartier_domicile_6`

Type of variable : ordinal

Question content : We're now going to ask you some questions about your living environment. More generally, these questions will help us gain a better understanding of your neighborhood.

We would like to know your perception of the neighborhood where your home is located. The neighborhood refers to the area within approximately a 10-15 minute walk from home. Using the following scale, please indicate your level of agreement with each of the statements. - Fast-food establishments are accessible within walking distance in the neighborhood.

Possible answers : - Strongly disagree: 1 - Somewhat disagree: 2 - Somewhat agree: 3 - Strongly agree: 4

Question 60

Question id : `quartier_domicile_7`

Type of variable : ordinal

Question content : We're now going to ask you some questions about your living environment. More generally, these questions will help us gain a better understanding of your neighborhood.

We would like to know your perception of the neighborhood where your home is located. The neighborhood refers to the area within approximately a 10-15 minute walk from home. Using the following scale, please indicate your level of agreement with each of the statements. - The neighborhood has green spaces conducive to relaxation or sporting activities.

Source : Coulombe S, Meunier S, Cloutier L, Auger N, Roy B, Tremblay G, de Montigny F, Gaboury I, Bernard F-O, Lavoie B, Dion H, Houle J. Health-promoting home and workplace neighborhoods: associations with multiple facets of men's health. Am J Mens Health 2017 Nov;11(6):1680–1691. PMID:29073845

Possible answers : - Strongly disagree: 1 - Somewhat disagree: 2 - Somewhat agree: 3 - Strongly agree: 4

Question 61

Question id : `quartier_domicile_8`

Type of variable : ordinal

Question content : We're now going to ask you some questions about your living environment. More generally, these questions will help us gain a better understanding of your neighborhood.

We would like to know your perception of the neighborhood where your home is located. The neighborhood refers to the area within approximately a 10-15 minute walk from home. Using the following scale, please indicate your level of agreement with each of the statements. - The neighborhood is safe enough for me to feel comfortable walking alone.

Source : Coulombe S, Meunier S, Cloutier L, Auger N, Roy B, Tremblay G, de Montigny F, Gaboury I, Bernard F-O, Lavoie B, Dion H, Houle J. Health-promoting home and workplace neighborhoods: associations with multiple facets of men's health. Am J Mens Health 2017 Nov;11(6):1680–1691. PMID:29073845

Possible answers : - Strongly disagree: 1 - Somewhat disagree: 2 - Somewhat agree: 3 - Strongly agree: 4

Question 62

Question id : `quartier_domicile_9`

Type of variable : ordinal

Question content : We're now going to ask you some questions about your living environment. More generally, these questions will help us gain a better understanding of your neighborhood.

We would like to know your perception of the neighborhood where your home is located. The neighborhood refers to the area within approximately a 10-15 minute walk from home. Using the following scale, please indicate your level of agreement with each of the statements. - There are places conducive to socializing in the neighborhood (e.g., public benches, picnic tables, outdoor terraces, community halls, etc.).

Source : Coulombe S, Meunier S, Cloutier L, Auger N, Roy B, Tremblay G, de Montigny F, Gaboury I, Bernard F-O, Lavoie B, Dion H, Houle J. Health-promoting home and workplace neighborhoods: associations with multiple facets of men's health. Am J Mens Health 2017 Nov;11(6):1680–1691. PMID:29073845

Possible answers : - Strongly disagree: 1 - Somewhat disagree: 2 - Somewhat agree: 3 - Strongly agree: 4

Question 63

Question id : `quartier_domicile_10`

Type of variable : ordinal

Question content : We're now going to ask you some questions about your living environment. More generally, these questions will help us gain a better understanding of your neighborhood.

We would like to know your perception of the neighborhood where your home is located. The neighborhood refers to the area within approximately a 10-15 minute walk from home. Using the following scale, please indicate your level of agreement with each of the statements. - The neighborhood is noisy, polluted and has a bad smell.

Possible answers : - Strongly disagree: 1 - Somewhat disagree: 2 - Somewhat agree: 3 - Strongly agree: 4

Question 64 (*)

Question id : `quartier_opportunite`

Type of variable : ordinal

Question content : My neighborhood offers many opportunities (infrastructure, sports and social activities, services/shops) to take care of my health.

Possible answers : - Strongly disagree: 1 - Disagree: 2 - Agree: 3 - Strongly agree: 4

Question 65

Question id : `fournisseurs_sante_1`

Type of variable : nominal_multiple

Question content : We're now going to ask you some questions about your use of healthcare services. More generally, these questions will help us gain a better understanding of your healthcare use.

Which of the following healthcare providers do you consult regularly? Check all that apply. Family doctor or general practitioner

Source : Statistique Canada. Enquête sur la santé dans les collectivités canadiennes (ESCC) - 2019. 2019. Available from:

Url link : Open link

Possible answers : Family doctor or general practitioner: 1

Question 66

Question id : `consult_sante`

Type of variable : nominal_single

Question content : In the past 12 months, have you seen or talked to a health professional about your emotional or mental health?

Source : Statistics Canada. Canadian Community Health Survey (CCHS) - 2015. 2016. Available from:

Url link : Open link

Possible answers : - Yes: 1 - No: 2

Question 67 (*)

Question id : `consult_who_1`

Type of variable : nominal_multiple

Question content : Whom did you see or talk to? Check all that apply. - Selected Choice Family doctor or general practitioner

Source : Statistics Canada. Canadian Community Health Survey (CCHS) - 2015. 2016. Available from:

Url link : Open link

Possible answers : Family doctor or general practitioner: 1

Question 68 (*)

Question id : `nb_friends_dispo`

Type of variable : integer

Question content : Please indicate the number of immediately available friends with whom you can talk (in person or over phone or text) frankly without having to watch what you say.

Source : Henderson S, Duncan-Jones P, Byrne DG, Scott R. Measuring social relationships: the interview schedule for social interaction. Psychological Medicine 1980;10(4):723–734.

Question 69 (*)

Question id : `issue_ai_data_3`

Type of variable : nominal_single

Question content : I agree that the government uses my numeric personal data, if it is for the public good.

Possible answers : - Strongly disagree: 1 - Somewhat disagree: 2 - Somewhat agree: 3 - Strongly agree: 4

Question 70

Question id : `friends_1_1`

Type of variable : nominal_multiple

Question content : How many friends from each of the following groups do you have? By "friends" we mean people you like and know well, but who are not members of your family. - native French speakers

Possible answers : - 1-2: 1 - 3-4: 2 - 5-6: 3 - 7 or more: 4

Question 71

Question id : `friends_2_1`

Type of variable : nominal_multiple

Question content : How many friends from each of the following groups do you have? By "friends" we mean people you like and know well, but who are not members of your family. - native English speakers

Possible answers : - 1-2: 1 - 3-4: 2 - 5-6: 3 - 7 or more: 4

Question 72

Question id : `friends_3_1`

Type of variable : nominal_multiple

Question content : How many friends from each of the following groups do you have? By "friends" we mean people you like and know well, but who are not members of your family. - people with another mother tongue

Possible answers : - 1-2: 1 - 3-4: 2 - 5-6: 3 - 7 or more: 4

Question 73

Question id : `ling_1`

Type of variable : float

Question content : On a scale of 0 to 10, how would you evaluate your skills in the following languages? - French

---

Question 74

Question id : `ling_2`

Type of variable : float

Question content : On a scale of 0 to 10, how would you evaluate your skills in the following languages? - English

---

14-item MHC-SF questions

Question `CSM_QA1_1`

Type of variable : ordinal

Question content : In the past month, how often have you felt... - Happy?

Source : Keyes CLM. Brief description of the mental health continuum short form (MHC-SF). 2009. Available from:

Url link : Open link

Possible answers : - Never: 1 - Once or twice: 2 - About once a week: 3 - About two or three times a week: 4 - Almost every day: 5 - Everyday: 6

Question `CSM_QA1_2`

Type of variable : ordinal

Question content : In the past month, how often have you felt... - Interested in life?

Source : Keyes CLM. Brief description of the mental health continuum short form (MHC-SF). 2009. Available from:

Url link : Open link

Possible answers : - Never: 1 - Once or twice: 2 - About once a week: 3 - About two or three times a week: 4 - Almost every day: 5 - Everyday: 6

Question CSM_QA1_3

Type of variable : ordinal

Question content : In the past month, how often have you felt... - Satisfied with your life?

Source : Keyes CLM. Brief description of the mental health continuum short form (MHC-SF). 2009. Available from:

Url link : Open link

Possible answers : - Never: 1 - Once or twice: 2 - About once a week: 3 - About two or three times a week: 4 - Almost every day: 5 - Everyday: 6

Question CSM_QA2_1

Type of variable : ordinal

Question content : Over the past month, how often have you felt... - that you had something important to contribute to society?

Source : Keyes CLM. Brief description of the mental health continuum short form (MHC-SF). 2009. Available from:

Url link : Open link

Possible answers : - Everyday: 1 - Almost every day: 2 - About two or three times a week: 3 - About once a week: 4 - Once or twice: 5 - Never: 6

Question CSM_QA2_2

Type of variable : ordinal

Question content : Over the past month, how often have you felt... - that you had a sense of belonging to a community (such as a social group, your neighborhood, your town, your school)?

Source : Keyes CLM. Brief description of the mental health continuum short form (MHC-SF). 2009. Available from:

Url link : Open link

Possible answers : - Everyday: 1 - Almost every day: 2 - About two or three times a week: 3 - About once a week: 4 - Once or twice: 5 - Never: 6

Question CSM_QA2_3

Type of variable : ordinal

Question content : Over the past month, how often have you felt... - that our society becomes a better place for people like you?

Source : Keyes CLM. Brief description of the mental health continuum short form (MHC-SF). 2009. Available from:

Url link : Open link

Possible answers : - Everyday: 1 - Almost every day: 2 - About two or three times a week: 3 - About once a week: 4 - Once or twice: 5 - Never: 6

Question CSM_QA2_4

Type of variable : ordinal

Question content : Over the past month, how often have you felt... - that people are fundamentally good?

Source : Keyes CLM. Brief description of the mental health continuum short form (MHC-SF). 2009. Available from:

Url link : Open link

Possible answers : - Everyday: 1 - Almost every day: 2 - About two or three times a week: 3 - About once a week: 4 - Once or twice: 5 - Never: 6

Question CSM_QA2_5

Type of variable : ordinal

Question content : Over the past month, how often have you felt... - that the way our society works makes sense to you?

Source : Keyes CLM. Brief description of the mental health continuum short form (MHC-SF). 2009. Available from:

Url link : Open link

Possible answers : - Everyday: 1 - Almost every day: 2 - About two or three times a week: 3 - About once a week: 4 - Once or twice: 5 - Never: 6

Question CSM_QA2_6

Type of variable : ordinal

Question content : Over the past month, how often have you felt… - that you like most aspects of your personality?

Source : Keyes CLM. Brief description of the mental health continuum short form (MHC-SF). 2009. Available from:

Url link : Open link

Possible answers : - Everyday: 1 - Almost every day: 2 - About two or three times a week: 3 - About once a week: 4 - Once or twice: 5 - Never: 6

Question CSM_QA2_7

Type of variable : ordinal

Question content : Over the past month, how often have you felt… - that you were good at handling the responsibilities of your day-to-day life?

Source : Keyes CLM. Brief description of the mental health continuum short form (MHC-SF). 2009. Available from:

Url link : Open link

Possible answers : - Everyday: 1 - Almost every day: 2 - About two or three times a week: 3 - About once a week: 4 - Once or twice: 5 - Never: 6

Question CSM_QA2_8

Type of variable : ordinal

Question content : Over the past month, how often have you felt… - that you had warm, trusting relationships with other people?

Source : Keyes CLM. Brief description of the mental health continuum short form (MHC-SF). 2009. Available from:

Url link : Open link

Possible answers : - Everyday: 1 - Almost every day: 2 - About two or three times a week: 3 - About once a week: 4 - Once or twice: 5 - Never: 6

Question CSM_QA2_9

Type of variable : ordinal

Question content : Over the past month, how often have you felt... - experiences that help you grow and become a better person?

Source : Keyes CLM. Brief description of the mental health continuum short form (MHC-SF). 2009. Available from:

Url link : Open link

Possible answers : - Everyday: 1 - Almost every day: 2 - About two or three times a week: 3 - About once a week: 4 - Once or twice: 5 - Never: 6

Question CSM_QA2_10

Type of variable : ordinal

Question content : Over the past month, how often have you felt... - able to think or express your own ideas and opinions?

Source : Keyes CLM. Brief description of the mental health continuum short form (MHC-SF). 2009. Available from:

Url link : Open link

Possible answers : - Everyday: 1 - Almost every day: 2 - About two or three times a week: 3 - About once a week: 4 - Once or twice: 5 - Never: 6

Question CSM_QA2_11

Type of variable : ordinal

Question content : Over the past month, how often have you felt... - that your life has a purpose or meaning?

Source : Keyes CLM. Brief description of the mental health continuum short form (MHC-SF). 2009. Available from:

Url link : Open link

Possible answers : - Everyday: 1 - Almost every day: 2 - About two or three times a week: 3 - About once a week: 4 - Once or twice: 5 - Never: 6
