# Supplementary material for: Explainable AI for Well-Being Prediction From Lifestyle Data: 2-Study Design
Source: JMIR Ment Health. 2026 May 8;13:e88750. doi: 10.2196/88750 (PMC13155431; doi:10.2196/88750)
Supplement: Multimedia Appendix 2 [file mental-v13-e88750-s002.docx]

## Technical details for the Machine Learning pipeline of Study 1

### Feature library for Study 1

From the 74 items retained for feature construction, see (Multimedia Appendix 1), each remaining item (i.e., question from the questionnaire of Study 1) was treated as an independent candidate feature. Numerical variables were retained in their original form, while categorical attributes were encoded using one-hot encoding. For categorical questions allowing multiple selections (nominal multiple), each response category was represented as a separate binary variable, whereas single-choice categorical variables (nominal single) were expanded into mutually exclusive binary indicators. No new features were derived by combining multiple attributes. Missing values were not imputed at this stage, as data imputation was handled within the cross-validation process to avoid data leakage. Following this preprocessing pipeline, the final feature library consisted of 214 features derived from 74 questionnaire items, capturing a broad range of lifestyle, social engagement, and sociodemographic characteristics available for supervised learning.

### Feature selection for Study 1

To ensure that the final model would remain interpretable and feasible for deployment within an online questionnaire, we applied a feature selection procedure designed to balance predictive accuracy with parsimony. The goal was to identify a subset of features, among the 214 features (i.e., corresponding to the 74 questions), that maximized explanatory power while keeping the questionnaire at a manageable length. We therefore set the questionnaire length to 20 questions as a compromise between retaining as much informative content as possible and limiting participant burden. Since a single question can map to multiple features, this constraint could still result in more than 20 features being retained. Feature importance scores were then computed to guide the selection under this constraint.

Feature importance was estimated using an Extreme Gradient Boosting (XGBoost) regressor, which has been shown to provide reliable and robust measures of variable importance in tabular prediction tasks [1]. Compared to linear or univariate statistical methods, XGBoost captures nonlinear relationships and higher-order feature interactions, providing a more comprehensive assessment of each feature’s contribution to the model’s predictive performance. The model was trained on the complete feature library, and feature importance was quantified based on each variable’s contribution to the reduction of prediction error across the ensemble of trees. The resulting importance scores were normalized using min–max scaling to facilitate comparison across variables.

To verify that limiting the questionnaire to 20 items constituted a reasonable compromise between predictive performance and respondent burden, we evaluated progressively larger subsets of the original questionnaire, which comprised 74 items (corresponding to 214 engineered features). Because overly long questionnaires can induce respondent fatigue and degrade data quality, we confined this analysis to configurations containing at most 40 items. We considered a series of candidate questionnaire lengths (5, 10, 15, 20, 25, 30, 35, and 40 questions) selected according to cumulative feature importance. For each candidate length, we computed the cumulative predictive contribution of the features associated with the corresponding set of unique questions, and quantified the marginal gain in explanatory power as the incremental increase in cumulative feature importance between successive lengths. We then identified a regime of diminishing returns, defined as the smallest questionnaire length beyond which the marginal gains decreased monotonically over at least three consecutive increments, indicating a sustained reduction in added predictive value. This analysis ensured that additional questions were only justified when they consistently improved model performance, and avoided unnecessary expansion of the questionnaire. On this basis, we confirmed that a moderate questionnaire length (corresponding to 20 items in our setting) achieved a favorable balance between performance and usability, and was therefore retained for subsequent analyses and online deployment.

### Model training for Study 1

We trained and evaluated multiple supervised learning models to predict participants’ well-being scores based on the selected features. Model evaluation followed a nested cross-validation procedure implemented in Python using scikit-learn. The dataset was first split into five folds (k = 5) to estimate out-of-sample predictive performance, ensuring sufficient training and test sample sizes (minimum of 30 observations per split). Within each training fold, a secondary three-fold cross-validation was used for hyperparameter optimization.

Within each cross-validation fold, models were implemented as part of a complete preprocessing and modeling pipeline comprising four stages: (1) imputation of missing values using either a mean imputer (SimpleImputer) or a k-nearest neighbors imputer (KNNImputer); (2) feature scaling using either a StandardScaler or a MinMaxScaler; (3) feature selection using an Extreme Gradient Boosting (XGBoost); and (4) model estimation. The model comparison included four families: (1) a mean regressor predicting the training-set mean (baseline), (2) a random regressor generating random predictions within the observed range (baseline), (3) a ridge regression model with the regularization parameter α tuned over {1, 2, 3, 4}, and (4) an XGBoost regressor tuned via grid search over maximum tree depth {3, 6, 9} and subsampling rate {0.5, 0.8}.

**Determining the Optimal Predictive Feature Set**

To identify which lifestyle and contextual factors best predict subjective well-being, we examined how the number of included predictors affected the model’s cumulative explanatory value. (Multimedia Appendix 2 Table 1) presents the trade-off between the number of features retained and their cumulative feature importance derived from the feature selection procedure. As expected, cumulative importance increased as more features were added, indicating that additional predictors contributed to the model’s explanatory capacity. However, the incremental gain associated with adding new features diminished after the initial configurations, suggesting that including larger sets of predictors yielded limited additional benefit.

Multimedia Appendix 2 Table 1. Cumulative and incremental feature importance values obtained from the feature selection procedure. The analysis considered subsets of increasing questionnaire length to identify the point at which additional questions provided diminishing predictive returns.^^[[1]](#footnote-1)^^

| Number of Questions | Number of Features | Cumulative Feature Importance | Incremental Gain |
| --- | --- | --- | --- |
| 5 | 6 | 4.81 | 0.00 |
| 10 | 15 | 8.67 | 3.86 |
| 15 | 23 | 10.89 | 2.22 |
| *20 (Selected)* | *36* | *13.90* | *3.02* |
| 25 | 46 | 15.88 | 1.98 |
| 30 | 55 | 17.36 | 1.48 |
| 35 | 62 | 18.41 | 1.05 |
| 40 | 73 | 19.88 | 1.46 |
| 45 | 82 | 20.95 | 1.08 |
| 50 | 98 | 22.64 | 1.69 |
| 55 | 106 | 23.42 | 0.77 |
| 60 | 124 | 24.95 | 1.53 |
| 65 | 137 | 25.91 | 0.96 |
| 70 | 160 | 27.35 | 1.44 |
| 74 | 214 | 28.49 | 1.15 |

The most substantial improvements in predictive contribution were observed when increasing the feature set from 5 to 10 predictors and again from 15 to 20 predictors. Beyond approximately 20 questions, incremental gains stabilized below two importance units, indicating diminishing returns relative to added model complexity. Based on this pattern, the set of 20 top-ranked questions (corresponding to 36 engineered features) was identified as providing the best balance between predictive performance and model parsimony.

The final set of 20 questions used in the model is presented with a star symbol in (Multimedia Appendix 1), together with their corresponding response formats. These items represent the subset of lifestyle, behavioral, and contextual variables that contributed most strongly to the prediction of well-being scores.

### Multicollinearity assessment

Multicollinearity among predictors can inflate variance in coefficient estimates and reduce model interpretability. Before training, interfeature collinearity was quantified using the variance inflation factor (VIF), ensuring that the selected features contributed independently to the prediction of well-being. As shown in (Multimedia Appendix 3), VIF values across all 36 features were tightly centered around 1, ranging between 1.01 and 1.28 (median approximately 1.09). These low values indicate minimal correlation among predictors and confirm that multicollinearity was not a concern in the model. Consequently, the stability and interpretability of the regression coefficients were preserved, supporting the robustness of the ridge regression results.

## References

1. Chen T, Guestrin C. XGBoost: a scalable tree boosting system. Proceedings of the 22nd ACM SIGKDD International Conference on Knowledge Discovery and Data Mining San Francisco, CA: ACM; 2016. p. 785–794. doi: 10.1145/2939672.2939785

1. The row in italics indicates the configuration selected for the final model. [↑](#footnote-ref-1)
