# Supplementary material for: Explainable AI for Well-Being Prediction From Lifestyle Data: 2-Study Design
Source: JMIR Ment Health. 2026 May 8;13:e88750. doi: 10.2196/88750 (PMC13155431; doi:10.2196/88750)

# Variance Inflation Factor (VIF) Analysis

To verify that the features included in the predictive model were not affected by multicollinearity, we computed the variance inflation factor (VIF) for each of the 36 selected predictors. VIF quantifies how strongly each feature is linearly correlated with the others; values above 5 are typically considered indicative of moderate multicollinearity, and values above 10 denote severe issues. As shown in the figure below, all VIF values ranged between 1.01 and 1.28, well below the conventional thresholds of 5 and 10 for moderate and severe multicollinearity, respectively, indicating negligible inter-feature correlation. These results confirm that the predictors contributed independently to the model and that coefficient estimates and feature importances remained stable and interpretable.


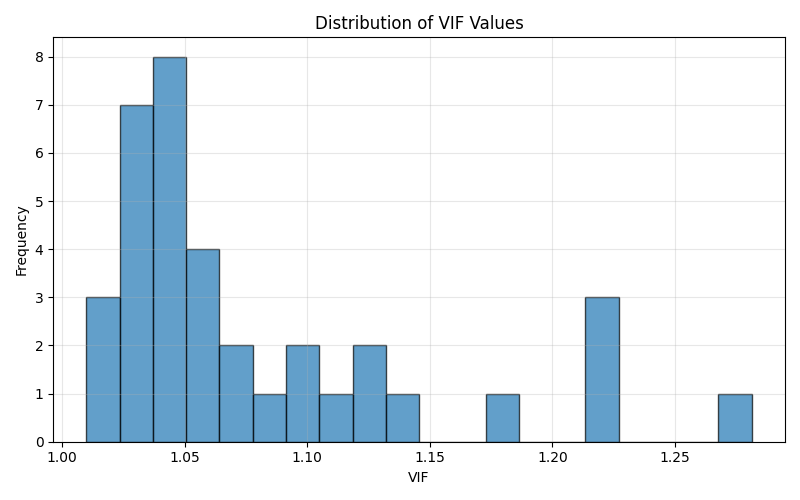

Supplement: Multimedia Appendix 3 [file mental-v13-e88750-s003.docx]
