# Supplementary material for: Explainable AI for Well-Being Prediction From Lifestyle Data: 2-Study Design
Source: JMIR Ment Health. 2026 May 8;13:e88750. doi: 10.2196/88750 (PMC13155431; doi:10.2196/88750)

**Ridge Regression Coefficients**

To interpret the contribution of individual predictors to the well-being model, we examined the standardized regression coefficients estimated by the ridge regression. Figure below displays the coefficients ordered from the strongest positive to the most negative associations with predicted well-being scores. Positive coefficients indicate that higher values on the corresponding feature are associated with greater predicted well-being, whereas negative coefficients denote the opposite relationship.


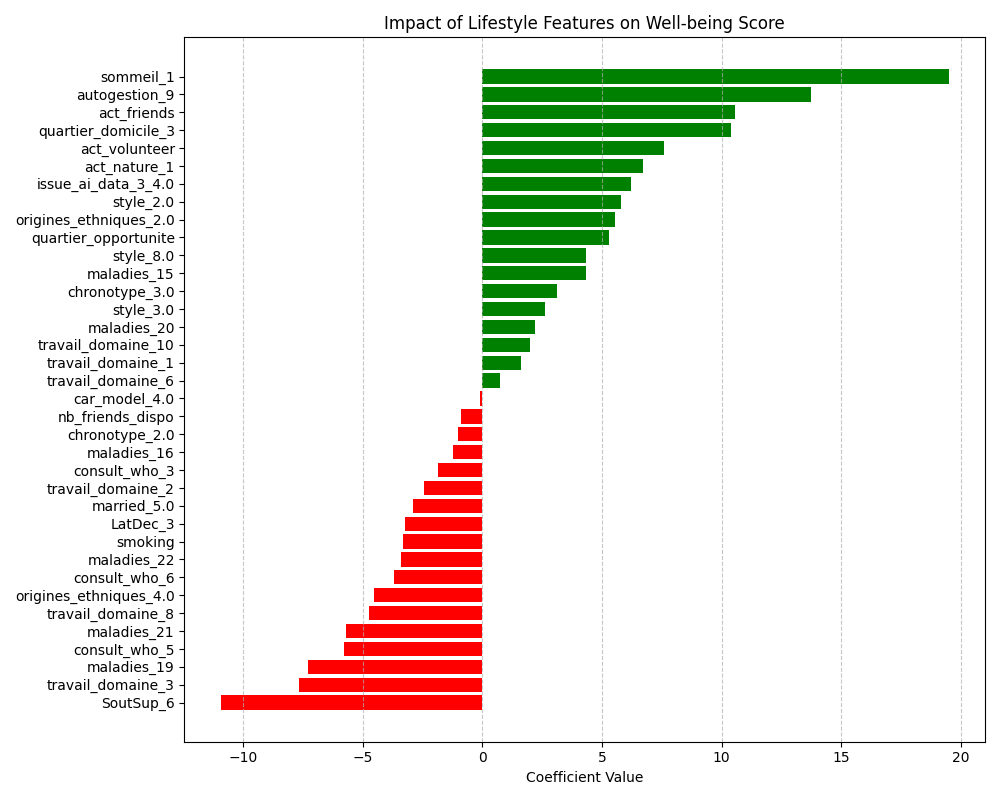

Supplement: Multimedia Appendix 4 [file mental-v13-e88750-s004.docx]
