# Supplementary material for: Explainable AI for Well-Being Prediction From Lifestyle Data: 2-Study Design
Source: JMIR Ment Health. 2026 May 8;13:e88750. doi: 10.2196/88750 (PMC13155431; doi:10.2196/88750)
Supplement: Multimedia Appendix 5 [file mental-v13-e88750-s005.pdf]

Questionnaire – Study 2

Source information The "Source" indicates the reference of the scale or instrument that informed each item, whether the item was inspired by that measure, adapted from it, or uses the same wording. Sources are provided only for items originating from validated instruments.

Sociodemographics

Question id : `sociodemo_01`

Form: `scroll`

Pilote ID: `genre`

Question content : What is your gender?

Possible answers : - Male (cisgender male) - Female (cisgender female) - Male (transgender male) - Female (transgender woman) - Non-binary - Queer - Agender - Other

---

Question id : `sociodemo_02`

Form: `scroll`

Question content : Were you born in Canada?

Possible answers : - Yes - No

Question id : `sociodemo_03`

Form: `scroll`

Pilote ID: `province`

Question content : In which province of Canada do you live?

Possible answers : - I don't live in Canada - Alberta - British Columbia - Prince Edward Island - Manitoba - New Brunswick - Nova Scotia - Nunavut - Ontario - Quebec - Saskatchewan - Newfoundland and Labrador - Northwest Territories - Yukon

---

Question id : `sociodemo_05`

Form: `scroll`

Pilote ID: `enfants`

Question content : How many children under the age of 18 live with you?

Possible answers : - 0 - 1 - 2 - 3 - 4 - 5 or more

Question id : `sociodemo_07`

Form: `scroll`

Pilote ID: `revenu`

Question content : Approximately, which of the following categories does your total household income, before taxes, fall into?

Possible answers : - No income - $1 to $30,000 - $30,001 to $60,000 - $60,001 to $90,000 - $90,001 to $110,000 - $110,001 to $150,000 - $150,001 to $200,000 - More than $200,000

Question id : `sociodemo_08`

Form: `scroll`

Pilote ID: `education`

Question content : What is the highest level of education that you have completed?

Possible answers : - No schooling - Elementary school - High school - College, CEGEP, or Classical College - Bachelor's degree - Master's degree - PhD

Question id : `sociodemo_09`

Form: `scroll`

Pilote ID: `age`

Question content : How old are you?

Possible answers : - 100+ - 18 - 19 - 20 - 21 - 22 - 23 - 24 - 25 - 26 - 27 - 28 - 29 - 30 - 31 - 32 - 33 - 34 - 35 - 36 - 37 - 38 - 39 - 40 - 41 - 42 - 43 - 44 - 45 - 46 - 47 - 48 - 49 - 50 - 51 - 52 - 53 - 54 - 55 - 56 - 57 - 58 - 59 - 60 - 61 - 62 - 63 - 64 - 65 - 66 - 67 - 68 - 69 - 70 - 71 - 72 - 73 - 74 - 75 - 76 - 77 - 78 - 79 - 80 - 81 - 82 - 83 - 84 - 85 - 86 - 87 - 88 - 89 - 90 - 91 - 92 - 93 - 94 - 95 - 96 - 97 - 98 - 99

---

20 predictor questions

Question id : `lifestyle_01`

Form: `checkbox`

Pilote ID: `travail_domaine`

Question content : Which one of the following categories best describes your field of employment?

Possible answers : - Management - Business, finance and administration - Natural and applied sciences and related fields - Health - Education, law and social, community and government services - Arts, Culture, Sport and Recreation - Sales and Service - Trades, transport, equipment operators and related occupations - Natural Resources, Agriculture and Related Production - Manufacturing and utilities - Other

---

Question id : `lifestyle_02`

Form: `scroll`

Pilote ID: `origines_ethniques`

Question content : Which of the following categories best describes you?

Possible answers : - White - Black - Indigenous - Asian - Hispanic - Arab - Other

---

Question id : `lifestyle_03`

Form: `scroll`

Pilote ID: `married`

Question content : What is your marital status?

Possible answers : - Single - Married - Common-law relationship - Widower/widow - Divorced/separated

---

Question id : `lifestyle_04`

Form: `scroll`

Pilote ID: `car_model`

Question content : Which of the following car models do you happen to use most often?

Possible answers : - 4x4 - Regular sedan or station wagon - Convertible or roadster - Pickup - Van or minivan - Luxury car (Mercedes, Porsche, etc.) - Sports car - Hybrid or electric car - SUV - Other - I do not have a car or I never use a car

---

Question id : `lifestyle_05`

Form: `likert`

Pilote ID: `smoking`

Question content : How often: do you smoke cigarettes and/or vape?

Possible answers : - Never - A few times a year - Once a month - Once a week - A few times a week - Once a day - More than once a day

---

Question id : `lifestyle_06`

Form: `likert`

Pilote ID: `act_friends`

Question content : How often: do you do activities with one or more friend(s)?

Possible answers : - Never - Almost never - Sometimes - Often - Very often

---

Question id : `lifestyle_07`

Form: `likert`

Pilote ID: `act_volunteer`

Question content : How often: do you volunteer or involve yourself in a cause?

Possible answers : - Never - Almost never - Sometimes - Often - Very often

---

Question id : `lifestyle_08`

Form: `likert`

Pilote ID: `act_nature_1`

Question content : How often: do you spend time in green or natural environments between May and September?

Possible answers : - Never - Almost never - Sometimes - Often - Very often

---

Question id : lifestyle_09

Form: scroll

Pilote ID: style

Question content : What is your clothing style?

Possible answers : - Hippie - Elegant - Classical - Casual - Formal - Punk - Rock - Sporty - Other

---

Question id : lifestyle_10

Form: checkbox

Pilote ID: maladies

Question content : A "long-term health problem" is expected to last or has already lasted 6 months or more and has been diagnosed by a health professional. Please tick the long-term health problem(s) you have.

Source : Statistics Canada. Canadian Community Health Survey (CCHS) - Mental Health. 2015. Available from:

Url link : Open link

Possible answers : - None of them - Diabetes - Cancer - Chronic fatigue syndrome - Chronic renal failure - Liver disease or gallbladder problems - Stomach or intestinal ulcers

---

Question id : lifestyle_11

Form: likert

Pilote ID: autogestion_9

Question content : How often have you followed a healthy diet in the past month?

Source : Coulombe S, Radziszewski S, Trépanier S-G, Provencher H, Roberge P, Hudon C, Meunier S, Provencher MD, Houle J. Mental health self-management questionnaire: Development and psychometric properties. Journal of Affective Disorders 2015 Aug 1;181:41–49. doi: 10.1016/j.jad.2015.04.007

Possible answers : - Never - Almost never - Sometimes - Often - Very often

---

Question id : `lifestyle_12`

Form: `cursor`

Pilote ID: `sommeil_1`

Question content : Please think about the quality of your sleep in the past seven days, such as how many hours of sleep you got, how easily you fell asleep, how often you woke up during the night (except to go to the bathroom), how often you woke up earlier than you had to in the morning, and how refreshing your sleep was. Please answer on a scale from 0 (terrible) to 10 (excellent). How would you rate your sleep quality over the past seven days?

Source : Snyder E, Cai B, DeMuro C, Morrison MF, Ball W. A new single-item sleep quality scale: results of psychometric evaluation in patients with chronic primary insomnia and depression. Journal of Clinical Sleep Medicine American Academy of Sleep Medicine; 2018;14(11):1849–1857. doi: 10.5664/jcsm.7478

Possible answers : - 0 - 1 - 2 - 3 - 4 - 5 - 6 - 7 - 8 - 9 - 10

Question id : `lifestyle_13`

Form: `scroll`

Pilote ID: `chronotype`

Question content : - Morning Type: High alertness in the morning, decreasing steadily throughout the day and lowest in the evening.;- Evening Type: Low alertness in the morning, increasing throughout the day and peaking in the evening.;- Highly active Type: Consistently high alertness throughout the day with little variation.;- Daytime sleep Type: High alertness in the morning and evening, with a dip during the daytime (likely due to daytime sleepiness).;- Diurnal Type: Peak alertness during the daytime, with lower levels in the morning and evening.;- Moderately active Type: Alertness stays moderate throughout the day with slight variation. Self-assess your chronotype by selecting the description that best represents the variation in your alertness levels throughout the day.

Source : Putilov AA, Sveshnikov DS, Puchkova AN, Dorokhov VB, Bakaeva ZB, Yakunina EB, . . . , Mairesse O. Single-Item Chronotyping (SIC), a method to self-assess diurnal types by using 6 simple charts. Personality and Individual Differences 2021 Jan 1;168:110353. doi: 10.1016/j.paid.2020.110353

Possible answers : - Morning - Evening - Highly active - Daytime sleep - Diurnal - Moderately active

---

Question id : `lifestyle_14`

Form: `likert`

Pilote ID: `LatDec_3`

Question content : To what extent do you agree with the following statements? In my working environment, I can decide when to take a break

Source : Gilbert-Ouimet M, Truchon M, Aubé K. Validation of the French version of the Questionnaire on Psychosocial Risks, Well-being, and Health at Work (QRBEST). Unpublished document. 2025.

Possible answers : - Strongly disagree - Somewhat disagree - Somewhat agree - Strongly agree

---

Question id : `lifestyle_15`

Form: `likert`

Pilote ID: `SoutSup_6`

Question content : To what extent do you agree with the following statements? In my working environment, there are times to discuss the difficulties involved in carrying out our task

Source : Gilbert-Ouimet M, Truchon M, Aubé K. Validation of the French version of the Questionnaire on Psychosocial Risks, Well-being, and Health at Work (QRBEST). Unpublished document. 2025.

Possible answers : - Strongly disagree - Somewhat disagree - Somewhat agree - Strongly agree

---

Question id : `lifestyle_16`

Form: `likert`

Pilote ID: `quartier_domicile_3`

Question content : To what extent do you agree with the following statements? In my neighborhood, the population is friendly (e.g., people smile, greet when they meet, or help each other, etc.)

Source : Coulombe S, Meunier S, Cloutier L, Auger N, Roy B, Tremblay G, de Montigny F, Gaboury I, Bernard F-O, Lavoie B, Dion H, Houle J. Health-promoting home and workplace neighborhoods: associations with multiple facets of men's health. Am J Mens Health 2017 Nov;11(6):1680–1691. PMID:29073845

Possible answers : - Strongly disagree - Somewhat disagree - Somewhat agree - Strongly agree

---

Question id : `lifestyle_17`

Form: `likert`

Pilote ID: `quartier_opportunite`

Question content : To what extent do you agree with the following statements? In my neighborhood, many opportunities (infrastructure, sports and social activities, services/shops) are offered to take care of my health

Possible answers : - Strongly disagree - Somewhat disagree - Somewhat agree - Strongly agree

---

Question id : `lifestyle_18`

Form: `checkbox`

Pilote ID: `consult_who`

Question content : In the past 12 months, have you seen or talked to a health professional about your emotional or mental health?

Source : Statistics Canada. Canadian Community Health Survey (CCHS) - 2015. 2016. Available from:

Url link : Open link

Possible answers : - No, I have not consulted a health professional regarding my emotional or mental health - Yes, I have consulted a family doctor or general practitioner - Yes, I have consulted a psychiatrist - Yes, I have consulted a psychologist - Yes, I have consulted a nurse - Yes, I have consulted a social worker or counselor - Yes, I have consulted an other health professional

---

Question id : `lifestyle_19`

Form: `cursor`

Pilote ID: `nb_friends_dispo`

Question content : Please indicate the number of immediately available friends with whom you can talk (in person or over phone or text) frankly without having to watch what you say.

Source : Henderson S, Duncan-Jones P, Byrne DG, Scott R. Measuring social relationships: the interview schedule for social interaction. Psychological Medicine 1980;10(4):723–734.

Possible answers : - 0 - 1 - 2 - 3 - 4 - 5 - 6 - 7 - 8 - 9 - 10+

Question id : `lifestyle_20`

Form: `likert`

Pilote ID: `issue_ai_data_3`

Question content : To what extent do you agree with the following statement? I agree that the government uses my numeric personal data, if it is for the public good

Possible answers : - Strongly disagree - Somewhat disagree - Somewhat agree - Strongly agree

---

Explanation stories

Depending on the experiment condition, the participant is redirected towards one of several explanation stories.

8-item explanation satisfaction and manipulation checks

Question id : `satis_01`

Form: `likert`

Question content : From the explanation, I understand how the algorithm works

Source : Hoffman RR, Mueller ST, Klein G, Litman J. Metrics for explainable AI: challenges and prospects. arXiv; 2019. doi: 10.48550/arXiv.1812.04608

Possible answers : - I disagree strongly - I disagree somewhat - I'm neutral about it - I agree somewhat - I agree strongly

---

Question id : `satis_02`

Form: `likert`

Question content : This explanation of how the algorithm works is satisfying

Source : Hoffman RR, Mueller ST, Klein G, Litman J. Metrics for explainable AI: challenges and prospects. arXiv; 2019. doi: 10.48550/arXiv.1812.04608

Possible answers : - I disagree strongly - I disagree somewhat - I'm neutral about it - I agree somewhat - I agree strongly

---

Question id : `satis_03`

Form: `likert`

Question content : This explanation of how the algorithm works has sufficient detail

Source : Hoffman RR, Mueller ST, Klein G, Litman J. Metrics for explainable AI: challenges and prospects. arXiv; 2019. doi: 10.48550/arXiv.1812.04608

Possible answers : - I disagree strongly - I disagree somewhat - I'm neutral about it - I agree somewhat - I agree strongly

---

Question id : `satis_04`

Form: `likert`

Question content : This explanation of how the algorithm works seems complete

Source : Hoffman RR, Mueller ST, Klein G, Litman J. Metrics for explainable AI: challenges and prospects. arXiv; 2019. doi: 10.48550/arXiv.1812.04608

Possible answers : - I disagree strongly - I disagree somewhat - I'm neutral about it - I agree somewhat - I agree strongly

---

Question id : `satis_05`

Form: `likert`

Question content : This explanation of how the algorithm works tells me how to use it

Source : Hoffman RR, Mueller ST, Klein G, Litman J. Metrics for explainable AI: challenges and prospects. arXiv; 2019. doi: 10.48550/arXiv.1812.04608

Possible answers : - I disagree strongly - I disagree somewhat - I'm neutral about it - I agree somewhat - I agree strongly

---

Question id : `satis_06`

Form: `likert`

Question content : This explanation of how the algorithm works is useful to my goals

Source : Hoffman RR, Mueller ST, Klein G, Litman J. Metrics for explainable AI: challenges and prospects. arXiv; 2019. doi: 10.48550/arXiv.1812.04608

Possible answers : - I disagree strongly - I disagree somewhat - I'm neutral about it - I agree somewhat - I agree strongly

---

Question id : `satis_07`

Form: `likert`

Question content : This explanation of the algorithm shows me how accurate the algorithm is

Source : Hoffman RR, Mueller ST, Klein G, Litman J. Metrics for explainable AI: challenges and prospects. arXiv; 2019. doi: 10.48550/arXiv.1812.04608

Possible answers : - I disagree strongly - I disagree somewhat - I'm neutral about it - I agree somewhat - I agree strongly

---

Question id : `satis_08`

Form: `likert`

Question content : This explanation lets me judge when I should trust and not trust the algorithm

Source : Hoffman RR, Mueller ST, Klein G, Litman J. Metrics for explainable AI: challenges and prospects. arXiv; 2019. doi: 10.48550/arXiv.1812.04608

Possible answers : - I disagree strongly - I disagree somewhat - I'm neutral about it - I agree somewhat - I agree strongly

---

Question id : `satis_09`

Form: `likert`

Question content : This explanation includes a chart

Possible answers : - I disagree strongly - I disagree somewhat - I'm neutral about it - I agree somewhat - I agree strongly

---

Question id : `satis_10`

Form: `likert`

Question content : This explanation uses descriptive text

Possible answers : - I disagree strongly - I disagree somewhat - I'm neutral about it - I agree somewhat - I agree strongly

---

Question id : `satis_11`

Form: `likert`

Question content : This explanation provides numerical details

Possible answers : - I disagree strongly - I disagree somewhat - I'm neutral about it - I agree somewhat - I agree strongly

---

Question id : `satis_12`

Form: `likert`

Question content : This explanation is interactive

Possible answers : - I disagree strongly - I disagree somewhat - I'm neutral about it - I agree somewhat - I agree strongly

---

Question id : `satis_13`

Form: `likert`

Question content : This explanation includes a population comparison

Possible answers : - I disagree strongly - I disagree somewhat - I'm neutral about it - I agree somewhat - I agree strongly

---

14-item well-being

Question id : `essaim_01`

Form: `likert`

Question content : In the past month, how often have you felt… Happy

Source : Keyes CLM. Brief description of the mental health continuum short form (MHC-SF). 2009. Available from:

Url link : Open link

Possible answers : - Never - Once or twice a month - About once a week - Two or three times a week - Almost every day - Every day

---

Question id : `essaim_02`

Form: `likert`

Question content : In the past month, how often have you felt… Interested in life

Source : Keyes CLM. Brief description of the mental health continuum short form (MHC-SF). 2009. Available from:

Url link : Open link

Possible answers : - Never - Once or twice a month - About once a week - Two or three times a week - Almost every day - Every day

---

Question id : `essaim_03`

Form: `likert`

Question content : In the past month, how often have you felt… Satisfied

Source : Keyes CLM. Brief description of the mental health continuum short form (MHC-SF). 2009. Available from:

Url link : Open link

Possible answers : - Never - Once or twice a month - About once a week - Two or three times a week - Almost every day - Every day

---

Question id : `essaim_04`

Form: `likert`

Question content : In the past month, how often have you felt… That you had something important to contribute to society

Source : Keyes CLM. Brief description of the mental health continuum short form (MHC-SF). 2009. Available from:

Url link : Open link

Possible answers : - Never - Once or twice a month - About once a week - Two or three times a week - Almost every day - Every day

---

Question id : `essaim_05`

Form: `likert`

Question content : In the past month, how often have you felt… That you belonged to a community (like a social group, your neighborhood, your city)

Source : Keyes CLM. Brief description of the mental health continuum short form (MHC-SF). 2009. Available from:

Url link : Open link

Possible answers : - Never - Once or twice a month - About once a week - Two or three times a week - Almost every day - Every day

---

Question id : `essaim_06`

Form: `likert`

Question content : In the past month, how often have you felt… That our society is becoming a better place for people

Source : Keyes CLM. Brief description of the mental health continuum short form (MHC-SF). 2009. Available from:

Url link : Open link

Possible answers : - Never - Once or twice a month - About once a week - Two or three times a week - Almost every day - Every day

---

Question id : `essaim_07`

Form: `likert`

Question content : In the past month, how often have you felt… That people are basically good

Source : Keyes CLM. Brief description of the mental health continuum short form (MHC-SF). 2009. Available from:

Url link : Open link

Possible answers : - Never - Once or twice a month - About once a week - Two or three times a week - Almost every day - Every day

---

Question id : `essaim_08`

Form: `likert`

Question content : In the past month, how often have you felt… That the way our society works makes sense to you

Source : Keyes CLM. Brief description of the mental health continuum short form (MHC-SF). 2009. Available from:

Url link : Open link

Possible answers : - Never - Once or twice a month - About once a week - Two or three times a week - Almost every day - Every day

---

Question id : `essaim_09`

Form: `likert`

Question content : In the past month, how often have you felt… That you liked most parts of your personality

Source : Keyes CLM. Brief description of the mental health continuum short form (MHC-SF). 2009. Available from:

Url link : Open link

Possible answers : - Never - Once or twice a month - About once a week - Two or three times a week - Almost every day - Every day

---

Question id : `essaim_10`

Form: `likert`

Question content : In the past month, how often have you felt… Good at managing the responsibilities of your daily life

Source : Keyes CLM. Brief description of the mental health continuum short form (MHC-SF). 2009. Available from:

Url link : Open link

Possible answers : - Never - Once or twice a month - About once a week - Two or three times a week - Almost every day - Every day

---

Question id : `essaim_11`

Form: `likert`

Question content : In the past month, how often have you felt… That you had warm and trusting relationships with others

Source : Keyes CLM. Brief description of the mental health continuum short form (MHC-SF). 2009. Available from:

Url link : Open link

Possible answers : - Never - Once or twice a month - About once a week - Two or three times a week - Almost every day - Every day

---

Question id : `essaim_12`

Form: `likert`

Question content : In the past month, how often have you felt… That you have experiences that challenge you to grow and become a better person

Source : Keyes CLM. Brief description of the mental health continuum short form (MHC-SF). 2009. Available from:

Url link : Open link

Possible answers : - Never - Once or twice a month - About once a week - Two or three times a week - Almost every day - Every day

---

Question id : `essaim_13`

Form: `likert`

Question content : In the past month, how often have you felt… Confident to think or express your own ideas and opinions

Source : Keyes CLM. Brief description of the mental health continuum short form (MHC-SF). 2009. Available from:

Url link : Open link

Possible answers : - Never - Once or twice a month - About once a week - Two or three times a week - Almost every day - Every day

---

Question id : `essaim_14`

Form: `likert`

Question content : In the past month, how often have you felt… That your life has a sense of direction or meaning to it

Source : Keyes CLM. Brief description of the mental health continuum short form (MHC-SF). 2009. Available from:

Url link : Open link

Possible answers : - Never - Once or twice a month - About once a week - Two or three times a week - Almost every day - Every day
