# Supplementary material for: Explainable AI for Well-Being Prediction From Lifestyle Data: 2-Study Design
Source: JMIR Ment Health. 2026 May 8;13:e88750. doi: 10.2196/88750 (PMC13155431; doi:10.2196/88750)

**Interfaces for the Explanation Modalities**

**Experimental condition**: 0 (control group)

**Modality**: No explanation


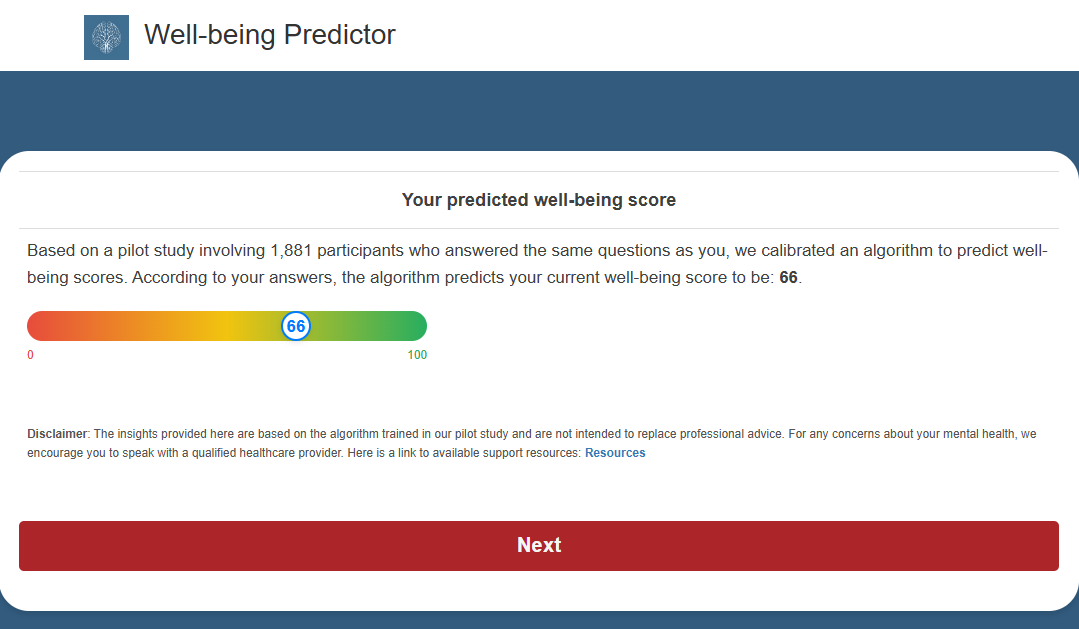


**Experimental condition**: 1

**Modality**: Contextual


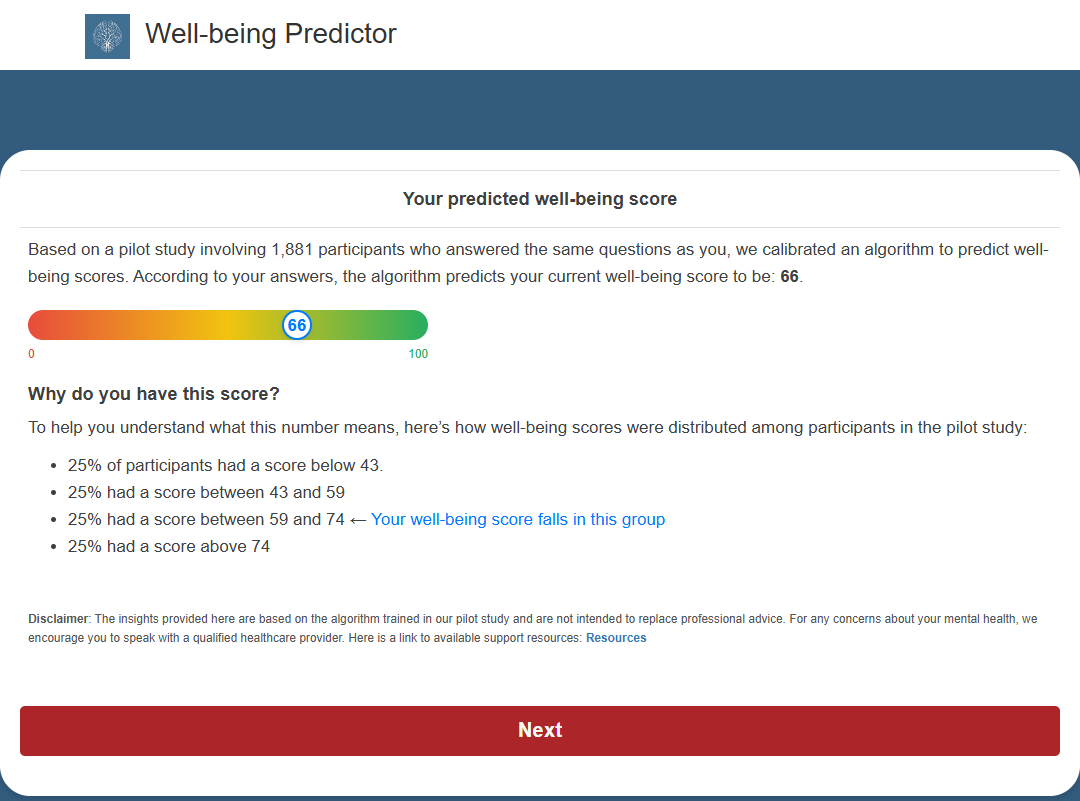


**Experimental condition**: 2

**Modality**: Quantitative


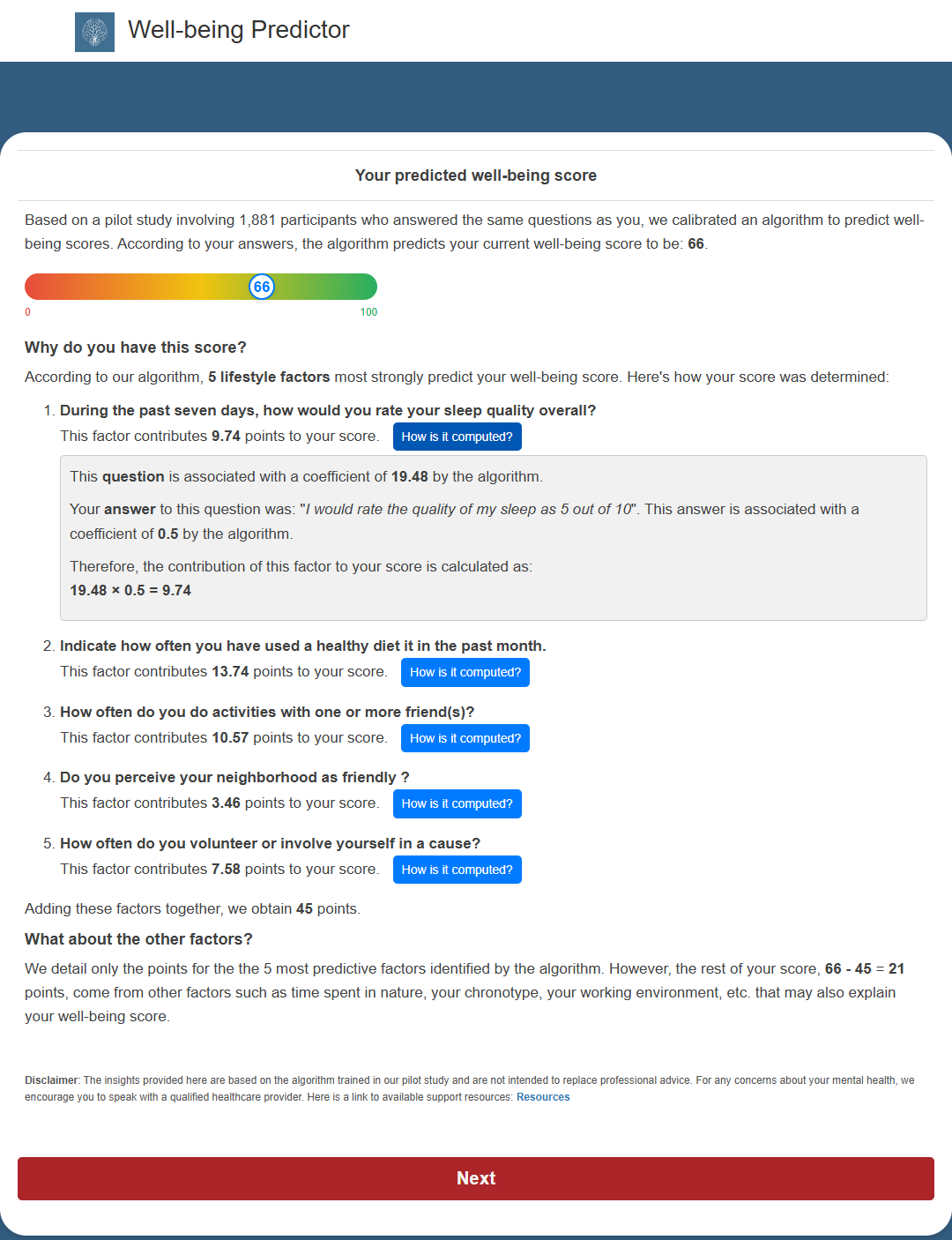


**Experimental condition**: 3

**Modality**: Textual


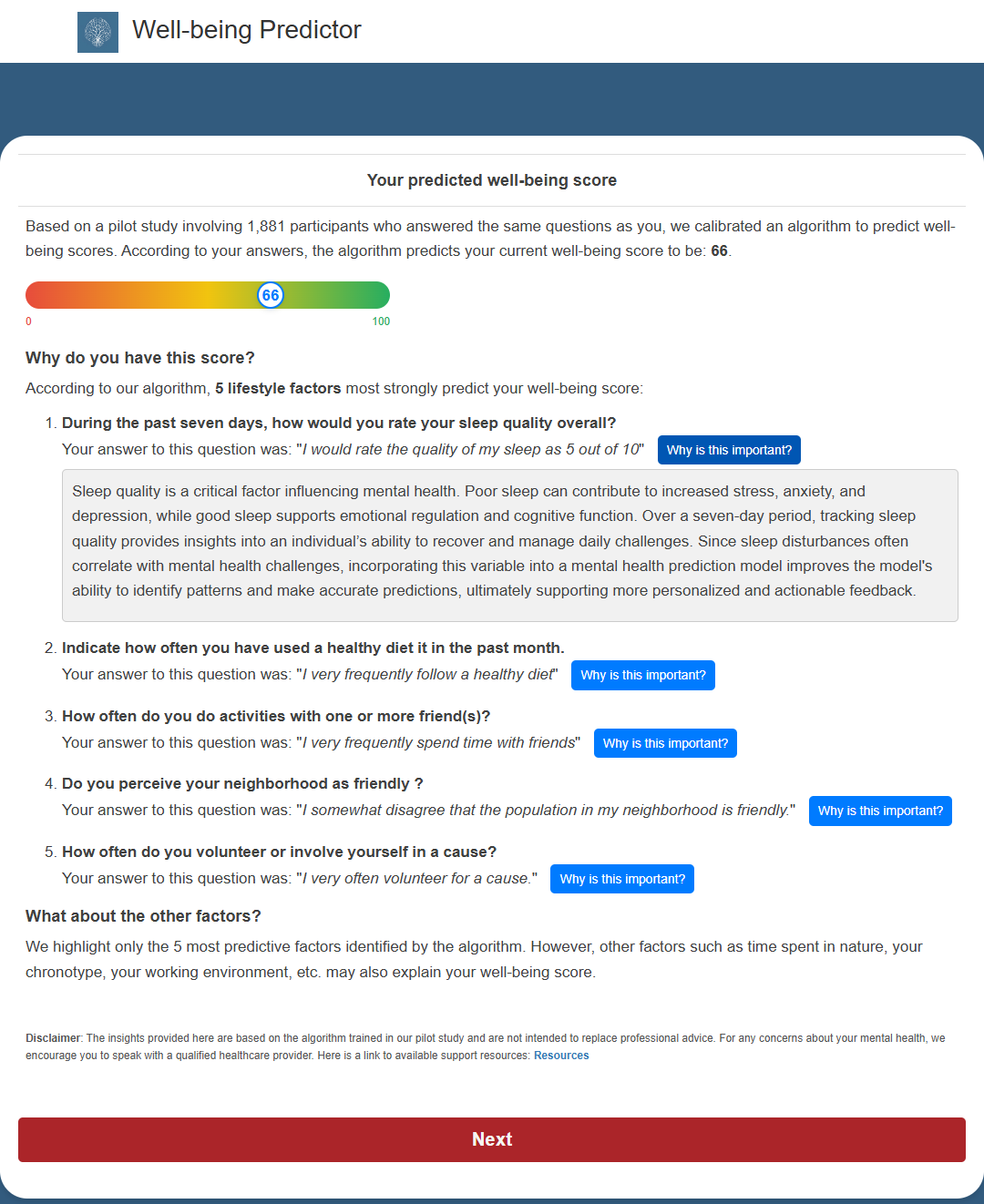


**Experimental condition**: 4

**Modality**: Visual


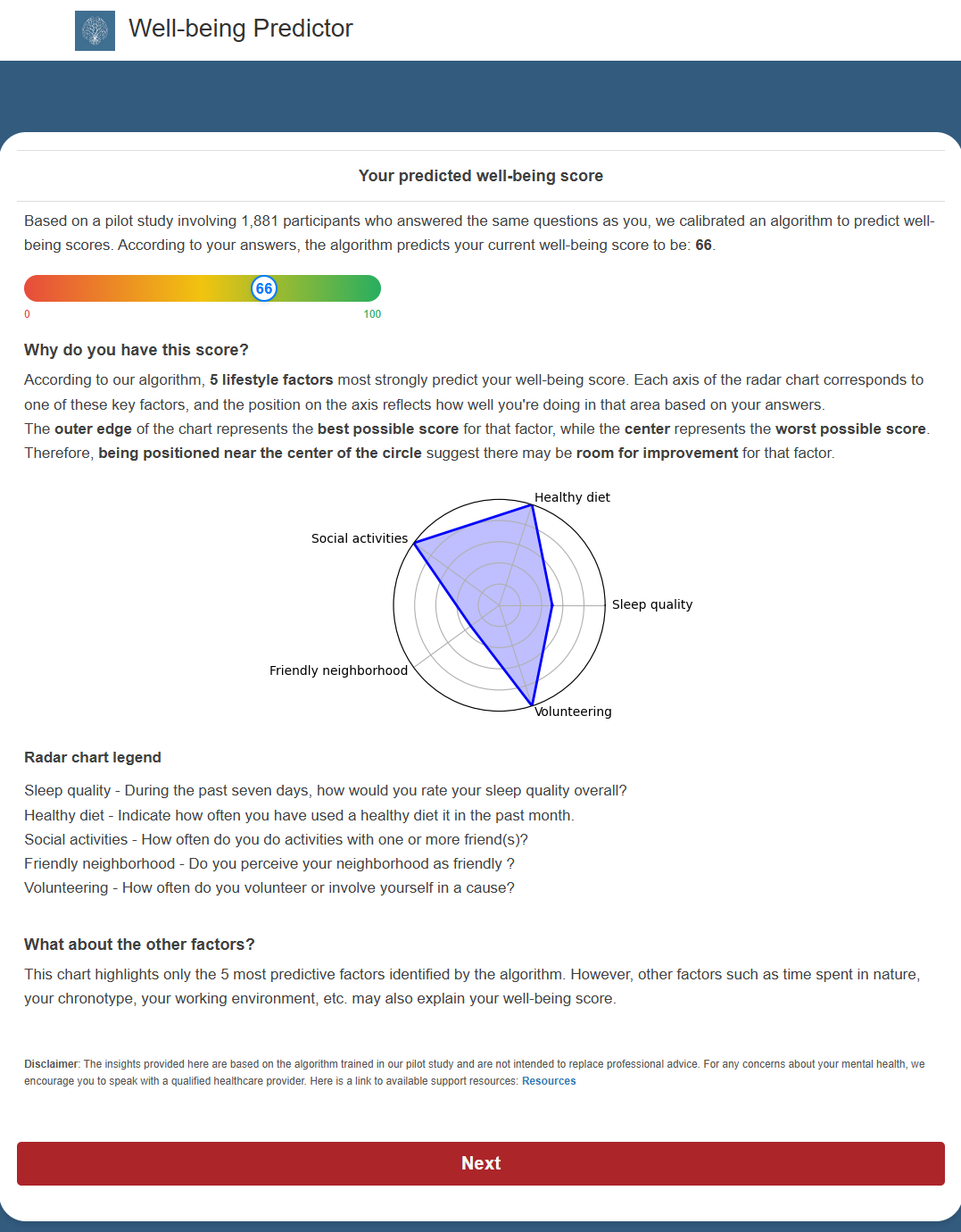


**Experimental condition**: 5

**Modality**: Interactive


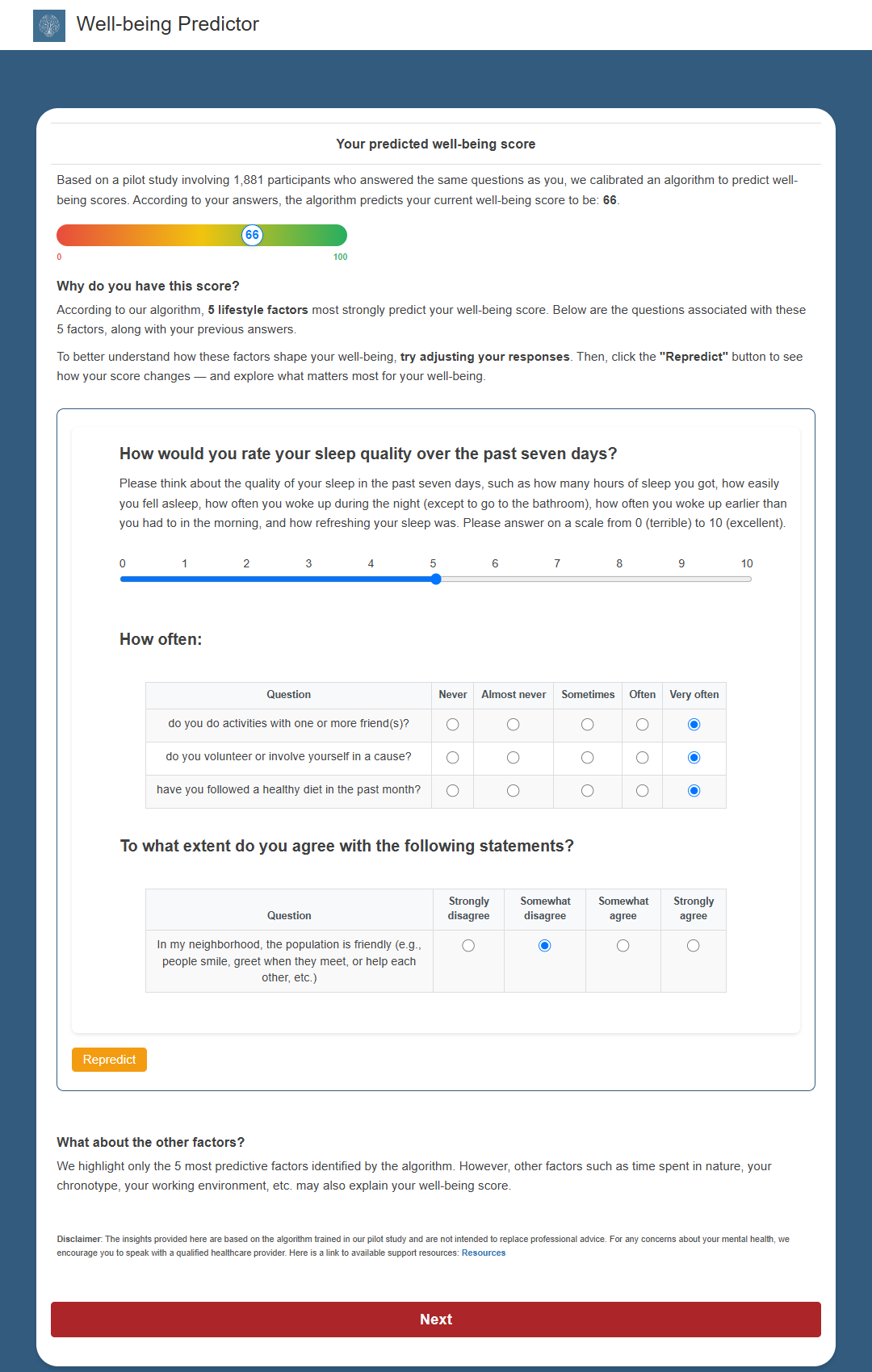

Supplement: Multimedia Appendix 6 [file mental-v13-e88750-s006.docx]
