# Supplementary material for: Explainable AI for Well-Being Prediction From Lifestyle Data: 2-Study Design
Source: JMIR Ment Health. 2026 May 8;13:e88750. doi: 10.2196/88750 (PMC13155431; doi:10.2196/88750)
Supplement: Multimedia Appendix 7 [file mental-v13-e88750-s007.docx]

## Technical details for explanation modalities design.

As a reminder, we rely on four explanation modalities for Study 2: quantitative, textual, visual, and interactive. The contextual condition differed conceptually and technically from the other four modalities, as it did not rely on model parameters but rather on population-level distributions derived from the pilot dataset.

The four explanation modalities (i.e., quantitative, textual, visual, and interactive) were derived directly from the parameters of the predictive model developed in Study 1. As established previously, the ridge regression model was retained for Study 2 based on its favorable balance between accuracy, simplicity, and interpretability. This model provides a set of regression coefficients that quantify the relative contribution of each predictor to the predicted well-being score. Because the model included 36 features (related to the 20 questions), presenting the full set of coefficients would have been cognitively demanding for participants and potentially detrimental to interpretability. To enhance clarity and ensure that explanations remained concise and actionable, we restricted the display to a subset of the most informative predictors. Specifically, only features associated with positive coefficients were retained for explanation. This choice was motivated by four considerations. First, limiting explanations to factors that positively influence well-being facilitates a coherent interpretative direction across modalities. Second, given the sensitive nature of the well-being domain, focusing on strengths rather than deficits avoids framing effects that could induce discomfort or negative emotions (for ex. blame or guilt). Third, positive coefficients align more naturally with the intervention-oriented purpose of the study, emphasizing opportunities for improvement rather than constraints. Finally, the model’s largest coefficients were predominantly positive in magnitude, see (Multimedia Appendix 4), further justifying this focus. Among the positively weighted predictors, the five features with the largest standardized coefficients were selected for explanation: sleep quality, autonomy at work, frequency of social activities with friends, satisfaction with one’s living environment, and volunteering behavior. These features, identified as the most influential determinants of predicted well-being (see Results of Study 1), served as the explanatory foundation for model-based conditions.

Unlike the other explanation modalities, which relied on language model–generated narratives to convey the model’s reasoning in natural language (i.e., quantitative, textual, and visual explanation modalities), the interactive explanation did not depend on any pre-written content

For the quantitative, textual, and visual explanation modalities, the explanation content was generated from the parameters of the ridge regression model retained from the first stage of the pipeline. Importantly, the phrasing and structure of each explanation modality were created once prior to the experiment using GPT-4o through structured prompting; no LLM calls occurred during the study. Thus, all participants assigned to the same explanation condition received the exact same explanation template, with only the numerical values inserted dynamically based on their individual responses. These personalized values were injected at runtime into pre-defined HTML templates using Jinja and Flask.

The following large language model (LLM) prompts were used to generate the content

of the quantitative, textual, and visual explanation modalities. All prompts were executed with ChatGPT (GPT-4o; OpenAI, 2024) and adapted to the experimental web platform. The resulting texts were integrated into the HTML templates rendered via Flask, using dynamic variable injection through the dictionary explain_dic.

### Quantitative Explanation

**LLM – Interaction 1**

I need to provide a high-quality explanation for users using a tool that predicts their mental health score based on their answers to lifestyle questions.

I would like to provide a quantitative explanation of the predicted score, showing numerically how the model combines the most informative factors.

The well-being score ranges from 0 to 100 (100 means excellent mental health, 0 means poor mental health). The prediction is produced by a ridge regression model trained on pilot data from 2,000 participants. The model predicts the well-being score as a weighted combination of the user’s responses to several lifestyle and socio-demographic variables, plus an intercept term.

For the current user, the five most influential features are automatically selected based on the largest positive coefficients. For each feature, both its standardized (scaled) value and raw response are available. The model predicts an overall well-being score, of which a portion is explained by these five factors.

In the context of explainable AI, please produce a concise paragraph that interprets how these factors combine to explain the predicted score, ensuring clarity for non-expert users.

**LLM – Interaction 2**

I now want the same text, formatted for inclusion in an HTML file between paragraph tags (<p>and</p>). The following variables are dynamically inserted at runtime via Flask’s render_template command using the dictionary explain_dic: predicted score, intermediate predicted score, number of informative features, and a dictionary mapping each feature ID to its question text, standardized value, coefficient, and explanatory text.

### Textual Explanation

**LLM – Interaction 1**

I need to provide a high-quality textual explanation for users of a tool predicting mental health from lifestyle data. The explanation should avoid numbers and mathematical notation, instead describing conceptually how the five most informative features contribute to the predicted score.

The model is a ridge regression predicting a 0–100 well-being score based on 20 questions; the top five predictors are selected by the largest positive coefficients. For the current user, the predicted score is computed automatically.

In the context of explainable AI, please write an accessible paragraph describing how these five lifestyle dimensions contribute to the individual’s well-being prediction.

**LLM – Interaction 2**

Format the explanation text in HTML between paragraph tags (<p>and</p>), where values are dynamically replaced via render_template. The dictionary explain_dic contains: predicted score, number of informative features, and a dictionary mapping each feature ID to its question text, detailed explanation, and coefficient (though coefficients are not displayed in this textual version).

**LLM – Interaction 3**

For each lifestyle question (for example, “During the past seven days, how would you rate your sleep quality overall?”), generate a short text paragraph explaining why this feature is relevant to mental health prediction. Output in plain text (.txt) format.

### Visual Explanation

**LLM – Interaction 1**

I need to provide a visual explanation accompanying a radar chart that displays the participant’s normalized responses for the five most influential features in the ridge regression model. The radar chart is already generated; please produce the textual summary that complements it.

The overall well-being score ranges from 0 to 100. The model predicts a given score, part of which is explained by the top five positive predictors. Each predictor corresponds to a lifestyle question (provided dynamically) with an associated regression coefficient and standardized response value.

In the context of explainable AI, write a concise narrative (without formulas) helping the user interpret the radar chart—emphasizing what the chart reveals about their strengths and areas for potential improvement.

**LLM – Interaction 2**

The final output is inserted in HTML between paragraph tags (<p>and</p>). Dynamic variables are passed through render_template as: predicted score, intermediate predicted score, number of informative features, and a dictionary mapping feature IDs to their text, coefficient, standardized value, and explanatory text.
