# Supplementary material for: Explainable AI for Well-Being Prediction From Lifestyle Data: 2-Study Design
Source: JMIR Ment Health. 2026 May 8;13:e88750. doi: 10.2196/88750 (PMC13155431; doi:10.2196/88750)
Supplement: Multimedia Appendix 8 [file mental-v13-e88750-s008.docx]

**Explanation satisfaction model results**

| Term | Estimate (B) | SE | t value | P value |
| --- | --- | --- | --- | --- |
| Intercept | 2.16 | 0.113 | 19.1 | P<.001 |
| **Explanation modality (reference: baseline)** |  |  |  |  |
| Contextual | 0.218 | 0.077 | 2.82 | P=.005 |
| Interactive | 0.914 | 0.076 | 12 | P<.001 |
| Quantitative | 0.782 | 0.077 | 10.1 | P<.001 |
| Textual | 0.85 | 0.076 | 11.2 | P<.001 |
| Visual | 0.915 | 0.077 | 11.9 | P<.001 |
| **Well-being variables** |  |  |  |  |
| Prediction error (\|pred – measured\|) | −0.003 | 0.002 | −1.42 | P=.156 |
| Measured well-being (MHC‑SF) | 0.007 | 0.001 | 5.96 | P<.001 |
| **Sociodemographic covariates** |  |  |  |  |
| Gender: Male (vs Female) | 0.108 | 0.045 | 2.41 | P=.016 |
| Not born in Canada (vs born) | 0.054 | 0.051 | 1.06 | P=.290 |
| Has ≥1 child (vs none) | 0.143 | 0.05 | 2.87 | P=.004 |
| Income: 60k–110k (vs ≤60k) | 0.14 | 0.056 | 2.5 | P=.013 |
| Income: 110k–150k (vs ≤60k) | −0.038 | 0.07 | −0.541 | P=.588 |
| Income: ≥150k (vs ≤60k) | 0.038 | 0.07 | 0.537 | P=.592 |
| Education: College/CEGEP (vs HS or less) | 0.031 | 0.077 | 0.4 | P=.690 |
| Education: University (vs HS or less) | −0.12 | 0.062 | −1.93 | P=.054 |
| Age (years) | −0.001 | 0.002 | −0.481 | P=.630 |

Linear model estimates (B) for satisfaction with the received explanation, with predictors including explanation modality (baseline reference), measured well-being (MHC-SF), prediction error (predicted−measured), and sociodemographic covariates. Reference levels were ≤US $60 000 for income and high school or less for education. Two-sided tests were used, with statistical significance evaluated at α=.05. Exact P values are reported in the table; significance codes were not used in accordance with JMIR guidelines.
